# Geometric Estimation of Multivariate Dependency

**DOI:** 10.3390/e21080787

**Published:** 2019-08-12

**Authors:** Salimeh Yasaei Sekeh, Alfred O. Hero

**Affiliations:** Department of Electrical Engineering and Computer Science, University of Michigan, Ann Arbor, MI 48109, USA

**Keywords:** Henze–Penrose mutual information, Friedman–Rafsky test statistic, geometric mutual information, convergence rates, bias and variance tradeoff, optimization, minimal spanning trees

## Abstract

This paper proposes a geometric estimator of dependency between a pair of multivariate random variables. The proposed estimator of dependency is based on a randomly permuted geometric graph (the minimal spanning tree) over the two multivariate samples. This estimator converges to a quantity that we call the geometric mutual information (GMI), which is equivalent to the Henze–Penrose divergence. between the joint distribution of the multivariate samples and the product of the marginals. The GMI has many of the same properties as standard MI but can be estimated from empirical data without density estimation; making it scalable to large datasets. The proposed empirical estimator of GMI is simple to implement, involving the construction of an minimal spanning tree (MST) spanning over both the original data and a randomly permuted version of this data. We establish asymptotic convergence of the estimator and convergence rates of the bias and variance for smooth multivariate density functions belonging to a Hölder class. We demonstrate the advantages of our proposed geometric dependency estimator in a series of experiments.

## 1. Introduction

Estimation of multivariate dependency has many applications in fields such as information theory, clustering, structure learning, data processing, feature selection, time series prediction, and reinforcement learning, see [1,2,3,4,5,6,7,8,9,10], respectively. It is difficult to accurately estimate the mutual information in high-dimensional settings, specially where the data is multivariate with an absolutely continuous density with respect to Lebesgue measure—the setting considered in this paper. An important and regular measure of dependency is the Shannon mutual information (MI), which has seen extensive use across many application domains. However, the estimation of mutual information can often be challenging. In this paper, we focus on a measure of MI that we call the Geometric MI (GMI). This MI measure is defined as the asymptotic large sample limit of a randomized minimal spanning tree (MST) statistic spanning the multivariate sample realizations. The GMI is related to a divergence measure called the Henze–Penrose divergence [11,12], and related to the multivariate runs test [13]. In [14,15], it was shown that this divergence measure can be used to specify a tighter bound for the Bayes error rate for testing if a random sample comes from one of two distributions the bound in [14,15] is tighter than previous divergence-type bounds such as the Bhattacharrya bound [16]. Furthermore, the authors of [17] proposed a non-parametric bound on multi-class classification Bayes error rate using a global MST graph.

Let X and Y be random variables with unknown joint density $f_{XY}$ and marginal densities $f_{X}$ and $f_{Y}$, respectively, and consider two hypotheses: $H_{0}$, X and Y are independent and $H_{1}$, X and Y are dependent,

$$H_{0}:f_{XY}=f_{X}f_{Y},\;\;\;\;\;\mathrm{versus}\;\;\;\;\;H_{1}:f_{XY}\neq f_{X}f_{Y}.$$

The GMI is defined as the Henze–Penrose divergence between $f_{XY}$ and $f_{X}f_{Y}$ which can be used as a dependency measure. In this paper, we prove that for large sample size *n* the randomized MST statistic spanning the original multivariate sample realizations and a randomly shuffled data set converges almost surely to the GMI measure. A direct implication of [14,15] is that the GMI provides a tighter bound on the Bayes misclassification rate for the optimal test of independence. In this paper, we propose an estimator based on a random permutation modification of the Friedman–Rafsky multivariate test statistic and show that under certain conditions the GMI estimator achieves the parametric mean square error (MSE) rate when the joint density is bounded and smooth. Importantly unlike other measures of MI, our proposed GMI estimator does not require explicit estimation of the joint and marginal densities.

Computational complexity is an important challenge in machine learning and data science. Most plug-in-based estimators, such as the kernel density estimator (KDE) or the K-nearest-neighbor (KNN) estimator with known convergence rate, require runtime complexity of $O(n^{2})$, which is not suitable for large scale applications. Noshad et al. proposed a graph theoretic direct estimation method based on nearest-neighbor ratios (NNR) [18]. The NNR estimator is based on *k*-NN graph and computationally more tractable than other competing estimators with complexity O(knlogn). The construction of the minimal spanning tree lies at the heart of the GMI estimator proposed in this paper. Since the GMI estimator is based on the Euclidean MST the dual-tree algorithm by March et al. [19] can be applied. This algorithm is based on the construction of Borůvka [20] and implements the Euclidean MST in approximately O(nlogn) time. In this paper, we experimentally show that for large sample size the proposed GMI estimator has faster runtime than the KDE plug-in method.

### 1.1. Related Work

Estimation of mutual information has a rich history. The most common estimators of MI are based on plug-in density estimation, e.g., using the histogram, kernel density or kNN density estimators [21,22]. Motivated by ensemble methods applied to divergence estimation [23,24], in [22] an ensemble method for combining multiple KDE bandwidths was proposed for estimating MI. Under certain smoothness conditions this ensemble MI estimator was shown to achieve parametric convergence rates.

Another class of estimators of multivariate dependency bypasses the difficult density estimation task. This class includes the statistically consistent estimators of Rényi-α and KL mutual information which are motivated by the asymptotic limit of the length of the KNN graph, [25,26] when joint density is smooth. The estimator of [27] builds on KNN methods for Rényi entropy estimation. The authors of [26], showed that when MI is large the KNN and KDE approaches are ill-suited for estimating MI since the joint density may be insufficiently smooth when there are strong dependencies. To overcome this issue an assumption on the smoothness of the density is required, see [28,29], and [23,24]. For all these methods, the optimal parametric rate of MSE convergence is achieved when the densities are either *d*, (d+1)/2 or d/2 times differentiable [30]. In this paper, we assume that joint and marginal densities are smooth in the sense that they belong to Hölder continuous classes of densities $\Sigma_{d}\left(\eta ,K\right)$, where the smoothness parameter η∈(0,1] and the Lipschitz constant K>0.

A MI measure based on the Pearson chi-square divergence was considered in [31] that is computational efficient and numerically stable. The authors of [27,32] used nearest-neighbor graph and minimal spanning tree approaches, respectively, to estimate Rényi mutual information. In [22], a non-parametric mutual information estimator was proposed using a weighted ensemble method with O(1/n) parametric convergence rate. This estimator was based on plug-in density estimation, which is challenging in high dimension.

Our proposed dependency estimator differs from previous methods in the following ways. First, it estimates a different measure of mutual information, the GMI. Second, instead of using the KNN graph the estimator of GMI uses a randomized minimal spanning tree that spans the multivariate realizations. The proposed GMI estimator is motivated by the multivariate runs test of Friedman and Rafsky (FR) [33] which is a multivariate generalization of the univariate Smirnov maximum deviation test [34] and the Wald-Wolfowitz [35] runs test in one dimension. We also emphasize that the proposed GMI estimator does not require boundary correction, in contrast to other graph-based estimators, such as, the NNR estimator [18], scalable MI estimator [36], or cross match statistic [37].

### 1.2. Contribution

The contribution of this paper has three components

(1) We propose a novel non-parametric multivariate dependency measure, referred to as geometric mutual information (GMI), which is based on graph-based divergence estimation. The geometric mutual information is constructed using a minimal spanning tree and is a function of the Friedman–Rafsky multivariate test statistic.
(2) We establish properties of the proposed dependency measure analogous to those of Shannon mutual information, such as, convexity, concavity, chain rule, and a type of data-processing inequality.
(3) We derive a bound on the MSE rate for the proposed geometric estimator. An advantage of the estimator is that it achieves the optimal MSE rate without the need for boundary correction, which is required for most plug-in estimators.

### 1.3. Organization

The rest of the paper is organized as follows. In Section 2, we define the geometric mutual information and establish some of its mathematical properties. In Section 2.2 and Section 2.3, we introduce a statistically consistent GMI estimator and derive a bound on its mean square error convergence rate. In Section 3 we verify the theory through experiments.

Throughout the paper, we denote statistical expectation by E and the variance by abbreviation Var. Bold face type indicates random vectors. All densities are assumed to be absolutely continuous with respect to non-atomic Lebesgue measure.

## 2. The Geometric Mutual Information (GMI)

In this section, we first review the definition of the Henze–Penrose (HP) divergence measure defined by Berisha and Hero in [13,14]. The Henze–Penrose divergence between densities *f* and *g* with domain $R^{d}$ for parameter p∈(0,1) is defined as follows (see [13,14,15]):

(1) $$D_{p}\left(f,g\right)=\frac{1}{4pq}\left[\int \frac{{\left(pf(x)-qg(x)\right)}^{2}}{pf(x)+qg(x)}\;dx-{\left(p-q\right)}^{2}\right],$$

where q=1−p. This functional is an *f*-divergence [38], equivalently, as an Ali-Silvey distance [39], i.e., it satisfies the properties of non-negativity, monotonicity, and joint convexity [15]. The measure (1) takes values in [0,1] and $D_{p}\left(f,g\right)=0$ if and only if f=g almost surely.

The mutual information measure is defined as follows. Let $f_{X}$, $f_{Y}$, and $f_{XY}$ be the marginal and joint distributions, respectively, of random vectors $X\in R^{d_{x}}$, $Y\in R^{d_{y}}$ where $d_{x}$ and $d_{y}$ are positive integers. Then by using (1), a Henze–Penrose generalization of the mutual information between X and Y, is defined by

(2) $$\begin{array}{cc} I_{p}\left(X;Y\right) & =D_{p}\left(f_{XY},f_{X}f_{Y}\right)\\ & =\frac{1}{4pq}\left[\;\int \int \;\frac{{\left(pf_{XY}\left(x,y\right)-qf_{X}\left(y\right)f_{Y}\left(y\right)\right)}^{2}}{pf_{XY}\left(x,y\right)+qf_{X}\left(x\right)f_{Y}\left(y\right)}\;dx\;dy-{\left(p-q\right)}^{2}\right]. \end{array}$$

We will show below that $I_{p}\left(X;Y\right)$ has a geometric interpretation in terms of the large sample limit of a minimal spanning tree spanning *n* sample realizations of the merged labeled samples X∪Y. Thus, we call $I_{p}\left(X;Y\right)$ the GMI between X and Y. The GMI satisfies similar properties to other definitions of mutual information, such as Shannon and Rényi mutual information. Recalling (3) in [14], an alternative form of $I_{p}$ is given by

(3) $$\begin{array}{c} I_{p}\left(X;Y\right)=1-A_{p}\left(X;Y\right)=\frac{u_{p}\left(X;Y\right)}{4pq}-\frac{{\left(p-q\right)}^{2}}{4pq}, \end{array}$$

where

(4) $$\begin{array}{c} A_{p}\left(X;Y\right)=\;\int \int \;\frac{f_{XY}\left(x,y\right)f_{X}\left(x\right)f_{Y}\left(y\right)}{pf_{XY}\left(x,y\right)+qf_{X}\left(x\right)f_{Y}\left(y\right)}\;dx\;dy=E_{XY}\left[{\left(p\;\frac{f_{XY}\left(X,Y\right)}{f_{X}\left(X\right)f_{Y}\left(Y\right)}+q\right)}^{-1}\right],\;\;\mathrm{and}\\ u_{p}\left(X;Y\right)=\;\int \int \;\frac{{\left(pf_{XY}\left(x,y\right)-qf_{X}\left(x\right)f_{Y}\left(y\right)\right)}^{2}}{pf_{XY}\left(x,y\right)+qf_{X}\left(x\right)f_{Y}\left(y\right)}\;dx\;dy=1-4pq\;A_{p}\left(X;Y\right). \end{array}$$

The function $A_{p}\left(X;Y\right)$ was defined in [13] and is called the geometric affinity between X and Y. The next subsection of the paper is dedicated to the basic inequalities and properties of the proposed GMI measure (2).

### 2.1. Properties of the Geometric Mutual Information

In this subsection we establish basic inequalities and properties of the GMI, $I_{p}$, given in (2). The following theorem shows that $I_{p}\left(X;Y\right)$ is a concave function in $f_{X}$ and a convex function in $f_{Y|X}$. The proof is given in Appendix A.1.

**Theorem** **1.** *Denote by ${\tilde{I}}_{p}\left(f_{XY}\right)$ the GMI $I_{p}\left(X;Y\right)$ when $X\in R^{d_{x}}$ and $Y\in R^{d_{y}}$ have joint density $f_{XY}$. Then the GMI satisfies*

(i) *Concavity in $f_{X}$: Let $f_{Y|X}$ be conditional density of Y given X and let $g_{X}$ and $h_{X}$ be densities on $R^{d_{x}}$. Then for $\lambda_{1},\lambda_{2}\in \left[0,1\right]$, $\lambda_{1}+\lambda_{2}=1$*
  (5) $${\tilde{I}}_{p}\left(\lambda_{1}f_{Y|X}g_{X}+\lambda_{2}f_{Y|X}h_{X}\right)\geq \lambda_{1}{\tilde{I}}_{p}\left(f_{Y|X}g_{X}\right)+\lambda_{2}{\tilde{I}}_{p}\left(f_{Y|X}h_{X}\right).$$
*The inequality is strict unless either $\lambda_{1}$ or $\lambda_{2}$ are zero or $h_{X}=g_{X}$.*
(ii) *Convexity in $f_{Y|X}$: Let $g_{Y|X}$ and $h_{Y|X}$ be conditional densities of Y given X and let $f_{X}$ be marginal density. Then for $\lambda_{1},\lambda_{2}\in \left[0,1\right]$, $\lambda_{1}+\lambda_{2}=1$*
  (6) $${\tilde{I}}_{p}\left(\lambda_{1}g_{Y|X}f_{X}+\lambda_{2}h_{Y|X}f_{X}\right)\leq \lambda_{1}{\tilde{I}}_{p}\left(g_{Y|X}f_{X}\right)+\lambda_{2}{\tilde{I}}_{p}\left(h_{Y|X}f_{X}\right).$$
*The inequality is strict unless either $\lambda_{1}$ or $\lambda_{2}$ are zero or $h_{Y|X}=g_{Y|X}$.*

The GMI, $I_{p}\left(X;Y\right)$, satisfies properties analogous to the standard chain rule and the data-processing inequality [40]. For random variables $X\in R^{d_{x}},\;Y\in R^{d_{y}}$, and $Z\in R^{d_{z}}$ with conditional density $f_{XY|Z}$ we define the conditional GMI

(7) $$\begin{array}{c} I_{p}\left(X;Y|Z\right)=E_{Z}\left[{\tilde{I}}_{p}\left(f_{XY|Z}\right)\right],\;\;\;\mathrm{where}\\ {\tilde{I}}_{p}\left(f_{XY|Z}\right)=\;1-\;\int \int \;\frac{f_{XY|Z}\left(x,y|z\right)f_{X|Z}\left(x|z\right)f_{Y|Z}\left(y|z\right)}{p\;f_{XY|Z}\left(x,y|z\right)+q\;f_{X|Z}\left(x|z\right)f_{Y|Z}\left(y|z\right)}\;dx\;dy. \end{array}$$

The next theorem establishes a relation between the joint and conditional GMI.

**Theorem** **2.** *For given d-dimensional random vector X with components $X_{1},X_{2},\cdots ,X_{d}$ and random variable Y,*

(8) $$I_{p}\left(X;Y\right)\geq I_{p}\left(X_{1};Y\right)-\sum_{i=1}^{d-1}\left(1-I_{p}\left(X_{i};Y|X^{i-1}\right)\right),$$

*where $X^{i}:=X_{1},X_{2},\cdots ,X_{i}$ and the conditional GMI $I_{p}\left(X_{i};Y|X^{i-1}\right)$ is defined in (7).*

For d=2 Theorem 2 reduces to

(9) $$I_{p}\left(X_{1},X_{2};Y\right)\geq I_{p}\left(X_{1};Y\right)-\left(1-I_{p}\left(X_{2};Y|X_{1}\right)\right),$$

Please note that when $\sum_{i=1}^{d-1}\left(1-I_{p}\left(X_{i};Y|X^{i-1}\right)\right)\geq 1$, the inequality (8) is trivial since $0\leq I_{p}\left(X_{1};Y\right)\leq 1$. The proof of Theorem 2 is given in Appendix A.2. Theorem 2 is next applied to the case where X and Y form a Markov chain. The proof of the following “leany” data-processing inequality (Proposition 1) is provided in Appendices section, Appendix A.3.

**Proposition** **1.** *Suppose random vectors X,Y,Z form a Markov chain denoted, X→Y→Z, in the sense that $f_{XYZ}=f_{X|Y}f_{Y|Z}f_{Z}$. Then for p∈(0,1)*

(10) $$I_{p}\left(Y;X\right)\geq I_{p}\left(Z;X\right)-{\left(p\;E_{XY}\left[\delta_{X,Y}\right]+\left(1-p\right)\right)}^{-1},$$

*where*

$$\delta_{X,Y}=\int \frac{f_{X|Y}\left(X|Y\right)\;f_{Z|Y}\left(z|Y\right)}{f_{X|Z}\left(X|z\right)}\;dz.$$

Furthermore, if both X→Y→Z and X→Z→Y together hold true, we have $I_{p}\left(Y;X\right)=I_{p}\left(Z;X\right)$.

The inequality in (10) becomes interpretable as the standard data-processing inequality $I_{p}\left(Y;X\right)\geq I_{p}\left(Z;X\right)$, when

$$E_{Z}\left[\frac{f(Z|Y)}{f(Z|X)}\right]=\infty ,$$

since

$$E_{XY}\left[\delta_{X,Y}\right]=E_{XY}\left(\frac{f(X|Y)}{f(X)}E_{Z}\left[\frac{f(Z|Y)}{f(Z|X)}\right]\right).$$

### 2.2. The Friedman–Rafsky Estimator

Let a random sample ${\left\{x_{i},y_{i}\right\}}_{i=1}^{n}$ from $f_{XY}\left(x,y\right)$ be available. Here we show that the GMI $I_{p}\left(X;Y\right)$ can be directly estimated without estimating the densities. The estimator is inspired by the MST construction of [33] that provides a consistent estimate of the Henze–Penrose divergence [14,15]. We denote by $z_{i}$ the *i*-th joint sample $x_{i},y_{i}$ and by $Z_{n}$ the sample set ${\left\{z_{i}\right\}}_{i=1}^{n}$. Divide the sample set $Z_{n}$ into two subsets $Z_{n^{\prime }}^{\prime }$ and $Z_{n^{\prime \prime }}^{\prime \prime }$ with the proportion $\alpha =n^{\prime }/n$ and $\beta =n^{\prime \prime }/n$, where α+β=1.

Denote by ${\tilde{Z}}_{n^{\prime \prime }}$ the set

$$\left\{\left(x_{i_{k}},y_{j_{k}}\right),\;k=1,\cdots ,n^{\prime \prime },\;\mathrm{selected}\;\mathrm{at}\;\mathrm{random}\;\mathrm{from}\;Z_{n^{\prime \prime }}^{\prime \prime }\right\}:$$

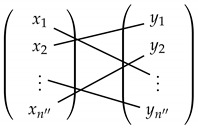


This means that for each $z_{ik}=\left(x_{ik},y_{ik}\right)\in Z_{n^{\prime \prime }}^{\prime \prime }$ given the first element $x_{ik}$ the second element $y_{ik}$ is replaced by a randomly selected $y\in {\left\{y_{jk}\right\}}_{j=1}^{n^{\prime \prime }}$. This results in a random shuffling of the binary relation relating $y_{ik}$ in $y_{jk}$. The estimator of $I_{p}\left(X;Y\right)$ is derived based on the Friedman–Rafsky (FR) multivariate runs test statistic [33] on the concatenated data set, $Z_{n^{\prime }}^{\prime }\cup {\tilde{Z}}_{n^{\prime \prime }}$. The FR test statistic is defined as the number of edges in the MST spanning the merged data set that connect a point in $Z_{n^{\prime }}^{\prime }$ to a point in ${\tilde{Z}}_{n^{\prime \prime }}$. This test statistic is denoted by $R_{n^{\prime },n^{\prime \prime }}:=R_{n^{\prime },n^{\prime \prime }}\left(Z_{n^{\prime }}^{\prime },{\tilde{Z}}_{n^{\prime \prime }}\right)$. Please note that since the MST is unique with probability one (under the assumption that all density functions are Lebesgue continuous) then all inter point distances between nodes are distinct. This estimator converges to $I_{p}\left(X;Y\right)$ almost surely as n→∞. The procedure is summarized in Algorithm 1.

**Algorithm 1:** MST-based estimator of GMI **Input:** Data set $Z_{n}:=\left\{{\left(x_{i},y_{i}\right)}_{i=1}^{n}\right\}$   1: Find $\tilde{\alpha }$ using arguments in Section 2.4   2: $n^{\prime }\leftarrow \tilde{\alpha }n$, $n^{\prime \prime }\leftarrow \left(1-\tilde{\alpha }\right)n$   3: Divide $Z_{n}$ into two subsets $Z_{n^{\prime }}^{\prime }$ and $Z_{n^{\prime \prime }}^{\prime \prime }$   4: ${\tilde{Z}}_{n^{\prime \prime }}\leftarrow \{{\left(x_{ik},y_{jk}\right)}_{k=1}^{n^{\prime \prime }}$: shuffle first and second elements of pairs in $Z_{n^{\prime \prime }}^{\prime \prime }\}$   5: $\hat{Z}\leftarrow Z_{n^{\prime }}^{\prime }\cup {\tilde{Z}}_{n^{\prime \prime }}^{\prime \prime }$   6: Construct MST on $\hat{Z}$   7: $R_{n^{\prime },n^{\prime \prime }}\leftarrow \#$ edges connecting a node in $Z_{n^{\prime }}^{\prime }$ to a node of ${\tilde{Z}}_{n^{\prime \prime }}$   8: ${\hat{I}}_{p}\leftarrow 1-R_{n^{\prime },n^{\prime \prime }}\frac{n^{\prime }+n^{\prime \prime }}{2n^{\prime }n^{\prime \prime }}$ **Output:**
${\hat{I}}_{p}$, where $p=\tilde{\alpha }$

Theorem 3 shows that the output in Algorithm 1 estimates the GMI with parameter p=α. The proof is provided in Appendix A.4.

**Theorem** **3.** *For given proportionality parameter α∈(0,1), choose $n^{\prime }$, $n^{\prime \prime }$ such that $n^{\prime }+n^{\prime \prime }=n$ and, as n→∞, we have $n^{\prime }/n\to \alpha$ and $n^{\prime \prime }/n\to \beta =1-\alpha$. Then*

(11) $$1-R_{n^{\prime },n^{\prime \prime }}\left(Z_{n^{\prime }}^{\prime },{\tilde{Z}}_{n^{\prime \prime }}\right)\;\frac{n}{2n^{\prime }\;n^{\prime \prime }}\to I_{\alpha }\left(X;Y\right),\;\;\;a.s.$$

**Figure 1:**
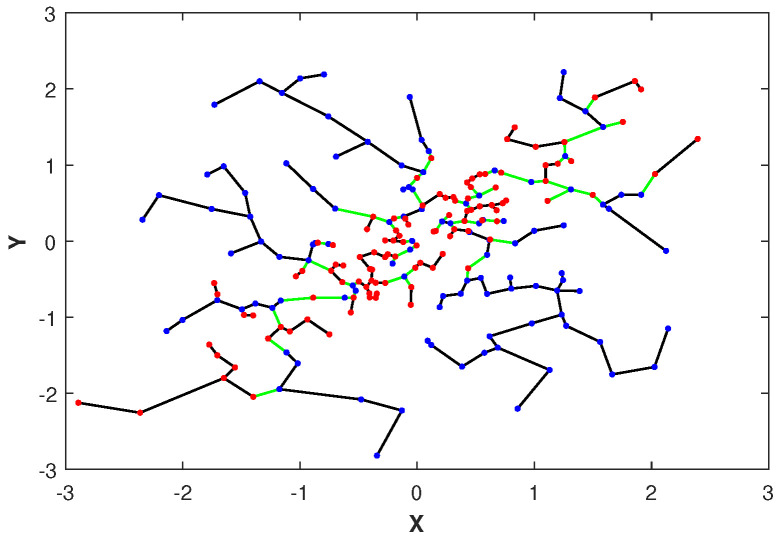
The MST and FR statistic of spanning the merged set of normal points when X and Y are independent (denoted in blue points) and when X and Y are highly dependent (denoted in red points). The FR test statistic is the number of edges in the MST that connect samples from different color nodes (denoted in green) and it is used to estimate the GMI $I_{p}$.

Please note that the asymptotic limit in (11) depends on the proportionality parameter α. Later in Section 2.4, we discuss the choice of an optimal parameter $\tilde{\alpha }$. In Figure 1, we illustrate the MST constructed over merged independent (ρ=0) and highly dependent (ρ=0.9) data sets drawn from two-dimensional normal distributions with correlation coefficients ρ. Notice that the edges of the MST connecting samples with different colors, corresponding to independent and dependent samples, respectively, are indicated in green. The total number of green edges is the FR test statistic $R_{n^{\prime },n^{\prime \prime }}\left(Z_{n^{\prime }}^{\prime },{\tilde{Z}}_{n^{\prime \prime }}\right)$.

### 2.3. Convergence Rates

In this subsection we characterize the MSE convergence rates of the GMI estimator of Section 2.2 in the form of upper bounds on the bias and the variance. This MSE bound is given in terms of the sample size *n*, the dimension *d*, and the proportionality parameter α. Deriving convergence rates for mutual information estimators has been of interest in information theory and machine learning [22,27]. The rates are typically derived in terms of a smoothness condition on the densities, such as the Hölder condition [41]. Here we assume $f_{X}$, $f_{Y}$ and $f_{XY}$ have support sets $S_{X}$, $S_{Y}$, and $S_{XY}:=S_{X}\times S_{Y}$, respectively, and are smooth in the sense that they belong to Hölder continuous classes of densities $\Sigma_{d}^{s}(\eta ,$ K), 0<η≤1 [42,43]:

**Definition** **1.** *(Hölder class): Let $X\subset R^{d}$ be a compact space. The Hölder class of functions $\Sigma_{d}\left(\eta ,K\right)$, with Hölder parameters η and K, consists of functions g that satisfy*

(12) $$\begin{array}{c} \left\{g:∥g\left(z\right)-p_{x}^{\left\lfloor \eta \right\rfloor }\left(z\right){∥}_{d}\leq K\;∥x-z{∥}_{d}^{\eta },\;\;x,\;z\in X\right\}, \end{array}$$

*where $p_{x}^{k}\left(z\right)$ is the Taylor polynomial (multinomial) of g of order k expanded about the point x and ⌊η⌋ is defined as the greatest integer strictly less than η.*

To explore the optimal choice of parameter α we require bounds on the bias and variance bounds, provided in Appendix A.5. To obtain such bounds, we will make several assumptions on the absolutely continuous densities $f_{X}$, $f_{Y}$, $f_{XY}$ and support sets $S_{X}$, $S_{Y}$, $S_{XY}$:

(**A**.1) Each of the densities belong to $\Sigma_{d}\left(\eta ,K\right)$ with smoothness parameters η and Lipschitz constant *K*.
(**A**.2) The volumes of the support sets are finite, i.e., $0<V\left(S_{X}\right)<\infty ,\;\;\;0<V\left(S_{Y}\right)<\infty$.
(**A**.3) All densities are bounded i.e., there exist two sets of constants $C_{X}^{L},C_{Y}^{L},C_{XY}^{L}$ and $C_{X}^{U},C_{Y}^{U},C_{XY}^{U}$ such that $0<C_{X}^{L}\leq f_{X}\leq C_{X}^{U}<\infty$, $0<C_{Y}^{L}\leq f_{Y}\leq C_{Y}^{U}<\infty$ and $0<C_{XY}^{L}\leq f_{XY}\leq C_{XY}^{U}<\infty$.

The following theorem on the bias follows under assumptions (**A**.1) and (**A**.3):

**Theorem** **4.** *For given α∈(0,1), β=1−α, d≥2, and 0<η≤1 the bias of the $R_{n^{\prime },n^{\prime \prime }}:=R_{n^{\prime },n^{\prime \prime }}\left(Z_{n^{\prime }}^{\prime },{\tilde{Z}}_{n^{\prime \prime }}\right)$ satisfies*

(13) $$\begin{array}{c} \left|\frac{E\left[R_{n^{\prime },n^{\prime \prime }}\right]}{n}-2\alpha \beta \;\int \int \;\frac{f_{XY}\left(x,y\right)f_{X}\left(x\right)f_{Y}\left(y\right)}{\alpha f_{XY}\left(x,y\right)+\beta f_{X}\left(x\right)f_{Y}\left(y\right)}\;dxdy\right|\\ \;\;\leq O\left(\max\left\{n^{-\eta^{2}/\left(d\left(1+\eta \right)\right)},\;{\left(\beta n\right)}^{-\eta /(1+\eta )},\;c_{d}n^{-1}\right\}\right), \end{array}$$

*where $c_{d}$ is the largest possible degree of any vertex of MST on $Z_{n^{\prime }}^{\prime }\cup {\tilde{Z}}_{n^{\prime \prime }}$. The explicit form of (13) is provided in Appendix A.5.*

Please note that according to Theorem 13 in [44], the constant $c_{d}$ is lower bounded by $\Omega \left(\sqrt{d}2^{n(1-H(\gamma ))}\right)$, $\gamma =2^{-d}$ and H(γ) is the binary entropy i.e.,

H(γ)=−γlogγ−(1−γ)log(1−γ).

A proof of Theorem 4 is given in Appendix A.5. The next theorem gives an upper bound on the variance of the FR estimator $R_{n^{\prime },n^{\prime \prime }}$. The proof of the variance result requires a different approach than the bias bound (the Efron–Stein inequality [45]). It is similar to arguments in ([46], Appendix A.3), and is omitted. In Theorem 5 we assume that the densities $f_{X}$, $f_{Y}$, and $f_{XY}$ are absolutely continuous and bounded (**A**.3).

**Theorem** **5.** *Given α∈(0,1), the variance of the estimator $R_{n^{\prime },n^{\prime \prime }}:=R_{n^{\prime },n^{\prime \prime }}\left(Z_{n^{\prime }}^{\prime },{\tilde{Z}}_{n^{\prime \prime }}\right)$ is bounded by*

(14) $$Var\left(\frac{R_{n^{\prime },n^{\prime \prime }}}{n}\right)\leq \frac{\left(1-\alpha \right)\;c_{d}}{n},\;\;\;\alpha =n^{\prime }/n,$$

*where $c_{d}$ is a constant depending only on the dimension d.*

### 2.4. Minimax Parameter α

Recall assumptions (**A**.1), (**A**.2), and (**A**.3) in Section 2.3. The constant α can be chosen to minimize the maximum the MSE converges rate where the maximum is taken over the space of Hölder smooth joint densities $f_{XY}$.

Throughout this subsection we use the following notations:

- $\epsilon_{XY}:=f_{XY}/f_{X}f_{Y}$,
- $C_{\epsilon }^{L}:=C_{XY}^{L}/C_{X}^{U}C_{Y}^{U}$ and $C_{\epsilon }^{U}:=C_{XY}^{U}/C_{X}^{L}C_{Y}^{L}$,
- $C_{n}:=C_{XY}^{L}\;n/2$,
- $\alpha_{0}^{L}:=\frac{2}{C_{n}}$ and $\alpha_{0}^{U}:=\min\left\{\frac{1}{4},\frac{1+1/C_{n}}{4+2C_{\epsilon }^{U}},1-n^{\eta /d-1}\right\}$, where η is the smoothness parameter,
- $l_{n}:=\left\lfloor n^{\eta /(d^{2}\left(1+\eta \right))}\right\rfloor$.

Now define ${\tilde{G}}_{\epsilon_{XY},n}^{\alpha ,\beta }\left(x,y\right)$ by

(15) $$\frac{\left(\epsilon_{XY}\left(x,y\right)+1/\left(\beta C_{n}\right)\right)\left(1+\epsilon_{XY}\left(xy\right)+1/\left(\beta C_{n}\right)\right)}{{\left(\alpha +\beta \epsilon_{XY}\left(x,y\right)\right)}^{2}},\;\;\;\beta =1-\alpha .$$

Consider the following optimization problem:

(16) $$\begin{array}{cc} \min_{\alpha }\;\;\max_{\epsilon_{XY}} & \tilde{\Delta }\left(\alpha ,\epsilon_{XY}\right)+c_{d}\left(1-\alpha \right)\;n^{-1}\\ \mathrm{subject}\;\mathrm{to} & C_{\epsilon }^{L}\leq \epsilon_{XY}\leq C_{\epsilon }^{U},\\ & \alpha_{0}^{L}\leq \alpha \leq \alpha_{0}^{U}, \end{array}$$

where

(17) $$\tilde{\Delta }\left(\alpha ,\epsilon_{XY}\right):=D\left(n,l_{n},d,\eta \right)+\tilde{D}\left(n,l_{n},d\right)C_{XY}^{U}{\;\int \int \;}_{S_{XY}}{\tilde{G}}_{\epsilon_{XY},n}^{\alpha ,\beta }\left(x,y\right)\;dxdy,$$

and

(18) $$\begin{array}{c} D\left(n,l_{n},d,\eta \right)=c_{2}l_{n}^{d}n^{-1}+c_{d}2^{d}n^{-1}+c^{\prime }l_{n}^{d}n^{-\eta /d}+cl_{n}^{d}n^{-1/d}+2c_{1}l_{n}^{d-1}n^{1/d-1}+c_{3}l_{n}^{-d\eta }, \end{array}$$

(19) $$\begin{array}{c} \tilde{D}\left(n,l_{n},d\right)=2+n^{-1}2c^{\prime \prime }\sum_{i=1}^{M}l_{n}\;l_{n}^{d}a_{i}^{-1}+n^{-3/2}2c_{1}^{\prime }\sum_{i=1}^{M}l_{n}\;l_{n}^{d/2}\sqrt{b_{i}}a_{i}^{2}\\ \;+n^{-1}\sum_{i=1}^{M}2n^{-3/2}l_{n}^{-d/2}\frac{\sqrt{b_{i}}}{a_{i}^{2}}{\left(na_{i}l_{n}^{d}+n^{2}a_{i}^{2}\right)}^{1/2}{\left(nb_{i}l_{n}^{d}+n^{2}b_{i}^{2}\right)}^{1/2}. \end{array}$$

Please note that in (18), $c,c^{\prime },c_{1},c_{2}$ are constants, and $c_{d}$ only depends on the dimension *d*. Also, in (19), $a_{i}$ and $b_{i}$ are constants. Let $\epsilon_{XY}^{*}$ be the optimal $\epsilon_{XY}$ i.e., $\epsilon_{XY}^{*}$ be the solution of the optimization problem (16). Set

(20) $$\begin{array}{c} \Xi \left(\alpha \right):=\frac{d}{d\alpha }\left(\tilde{\Delta }\left(\alpha ,\epsilon_{XY}^{*}\right)+c_{d}\left(1-\alpha \right)\;n^{-1}\right), \end{array}$$

such that $\tilde{\Delta }\left(\alpha ,\epsilon_{XY}^{*}\right)$ is (17) when $\epsilon_{XY}=\epsilon_{XY}^{*}$. For $\alpha \in [\alpha_{0}^{L},\alpha_{0}^{U}]$, the optimal choice of $\epsilon_{XY}$ in terms of maximizing the MSE is $\epsilon_{XY}^{*}=C_{\epsilon }^{U}$ and the saddle point for the parameter α, denoted by $\tilde{\alpha }$, is given as follows:

- $\tilde{\alpha }=\alpha_{0}^{U}$, if $\Xi (\alpha_{0}^{U})<0$.
- $\tilde{\alpha }=\alpha_{0}^{L}$, if $\Xi (\alpha_{0}^{L})>0$.
- $\tilde{\alpha }=\Xi^{-1}\left(0\right)$, if $\alpha_{0}^{L}\leq \Xi^{-1}\left(0\right)\leq \alpha_{0}^{U}$.

Further details are given in Appendix A.6.

## 3. Simulation Study

In this section, numerical simulations are presented that illustrate the theory in Section 2. We perform multiple experiments to demonstrate the utility of the proposed GMI estimator of the HP-divergence in terms of the dimension *d* and the sample size *n*. Our proposed MST-based estimator of the GMI is compared to density plug-in estimators of the GMI, in particular the standard KDE density plug-in estimator of [22], where the convergence rates of Theorems 4 and 5 are validated. We use multivariate normal simulated data in the experiments. In this section, we also discuss the choice of the proportionality parameter α and compare runtime of the proposed GMI estimator approach with KDE method.

**Figure 2:**
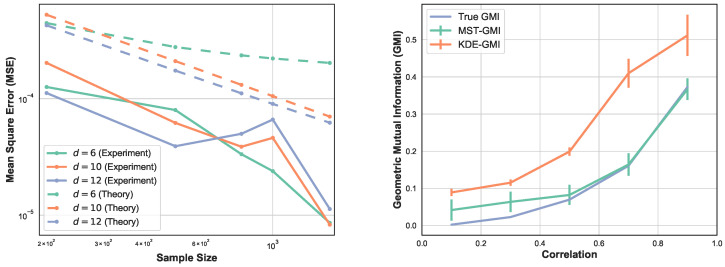
(**left**) Log–log plot of theoretical and experimental MSE of the proposed MST-based GMI estimator as a function of sample size *n* for d=6,10,12 and fixed smoothness parameter η. (**right**) The GMI estimator was implemented using two approaches, Algorithm 1 and KDE method where the KDE-GMI used KDE density estimators in the formula (2). In this experiment, samples are generated from the two-dimensional normal distribution with zero mean and covariance matrix (21) for various value of ρ∈[0.1,0.9].

Here we perform four sets of experiments to illustrate the proposed GMI estimator. For the first set of experiments the MSE of the GMI estimator in Algorithm 1 is shown in Figure 2-left. The samples were drawn from *d*-dimensional normal distribution, with various sample sizes and dimensions d=6,10,12. We selected the proportionality parameter α=0.3 and computed the MSE in terms of the sample size *n*. We show the log–log plot of MSE when *n* varies in [100,1500]. Please note that the empirically optimal proportion α depends on *n*, so to avoid the computational complexity we fixed α for this experiment. The experimental result shown in Figure 2-left validates the theoretical MSE growth rates derived from (13) and (14), i.e., decreasing sub-linearly in *n* and increasing exponentially in *d*.

In Figure 2-right, we compare the proposed MST-based GMI estimator with the KDE-GMI estimator [22]. For the KDE approach, we estimated the joint and marginal densities and then plugged them into the proposed expression (2). The bandwidth *h* used for the KDE plug-in estimator was set as $h=n^{-1/(d+1)}$. The choice of *h* minimizes the bound on the MSE of the plug-in estimator. We generated data from the two-dimensional normal distribution with zero mean and covariance matrix

(21) $$\left(\begin{array}{cc} 1 & \rho \\ \rho & 1 \end{array}\right).$$

The coefficient ρ is varied in range [0.1,0.9]. The true GMI was computed by the Monte Carlo approximation to the integral (2). Please note that as ρ increases, the MST-GMI outperforms the KDE-GMI approach. In this set of experiments α=0.6.

**Figure 3:**
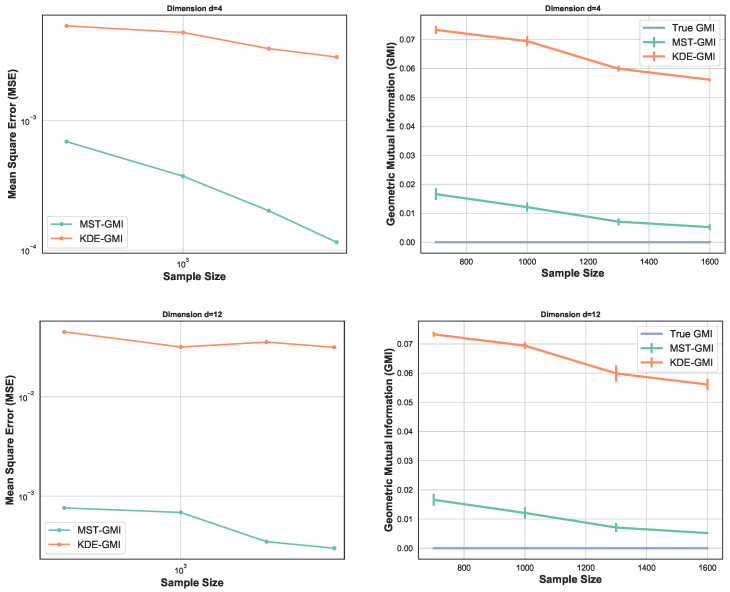
MSE log–log plots as a function of sample size *n* (**left**) for the proposed MST-GMI estimator (“Estimated GMI”) and the standard KDE-GMI plug-in estimator of GMI. The right column of plots correspond to the GMI estimated for dimension d=4 (**top**) and d=12 (**bottom**). In both cases the proportionality parameter α is 0.5. The MST-GMI estimator in both plots for sample size *n* in [700,1600] outperforms the KDE-GMI estimator, especially for larger dimensions.

Figure 3 again compares the MST-GMI estimator with the KDE-GMI estimator. samples are drawn from the multivariate standard normal distribution with dimensions d=4 and d=12. In both cases the proportionality parameter α=0.5. The left plots in Figure 3 show the MSE (100 trials) of the GMI estimator implemented with an KDE estimator (with bandwidth as in Figure 2 i.e., $h=n^{-1/(d+1)}$) for dimensions d=4,12 and various sample sizes. For all dimensions and sample sizes the MST-GMI estimator also outperforms the plug-in KDE-GMI estimator based on the estimated log–log MSE slope given in Figure 3 (left plots). The right plots in Figure 3 compares the MST-GMI with the KDE-GMI. In this experiment, the error bars denote standard deviations with 100 trials. We observe that for higher dimension d=12 and larger sample size *n*, the KDE-GMI approaches the true GMI at a slower rate than the MST-GMI estimator. This reflects the power of the proposed graph-based approach to estimating GMI.

**Figure 4:**
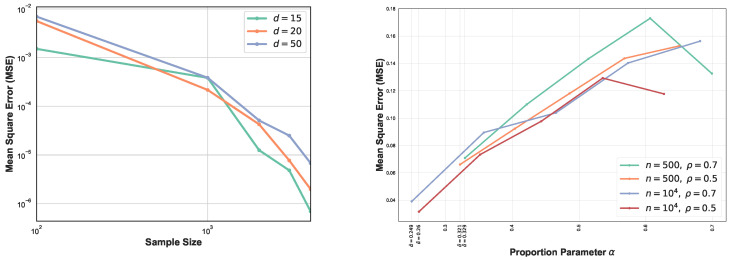
MSE log–log plots as a function of sample size *n* for the proposed FR estimator. We compare the MSE of our proposed FR estimator for various dimensions d=15,20,50 (**left**). As *d* increases, the blue curve takes larger values than green and orange curves i.e., MSE increases as *d* grows. However, this is more evidential for large sample size *n*. The second experiment (**right**) focuses on optimal proportion α for $n=500,10^{4}$ and ρ=0.7,0.5. $\tilde{\alpha }$ is the optimal α for $\alpha \in [\alpha_{0}^{L},\alpha_{0}^{U}]$.

The comparison between MSEs for various dimension *d* is shown in Figure 4 (left). This experiment highlights the impact of higher dimension on the GMI estimators. As expected, for larger sample size *n*, MSE decreases while for higher dimension it increases. In this setting, we have generated samples from standard normal distribution with size $n\in [10^{2},4\times 10^{3}]$ and α=0.5. From Figure 4 (left) we observe that for larger sample size, MSE curves are ordered based on their corresponding dimensions. Results in Section 2.4 strongly depend on the lower bounds $C_{X}^{L},\;C_{Y}^{L},\;C_{XY}^{L}$ and upper bounds $C_{X}^{U},\;C_{Y}^{U},\;C_{XY}^{U}$ and provide optimal parameter α in the range $\left[\alpha_{0}^{L},\alpha_{0}^{U}\right]$, therefore in the experiment section we only analyze one case where the lower bounds $C_{X}^{L},\;C_{Y}^{L},\;C_{XY}^{L}$ and upper bounds $C_{X}^{U},\;C_{Y}^{U},\;C_{XY}^{U}$ are known and the optimal α becomes $\alpha_{0}^{L}$. Figure 4 (right) illustrates the MSE vs proportion parameter α when $n=500,10^{4}$ samples are generated from truncated normal distribution with ρ=0.7,0.5. First, following Section 2.4, we compute the bound $\left[\alpha_{0}^{L},\alpha_{0}^{U}\right]$ and then derive the optimal α in this range. Therefore, each experiment with different sample size and ρ provides different range $\left[\alpha_{0}^{L},\alpha_{0}^{U}\right]$. We observe that the MSE does not appeared a monotonic function in α and its behavior strongly depends on sample size *n*, *d*, and density functions’ bounds. Additional study of the dependency is described in Appendix A.6. In this set of experiments $\Xi (\alpha_{0}^{L})>0$, therefore following the results in Section 2.4, we have $\tilde{\alpha }=\alpha_{0}^{L}$. In this experiment the optimal value of α is always the lower bound $\alpha_{0}^{L}$ and indicated in the Figure 4 (right).

The parameter α is studied further for three scenarios where the lower bounds $C_{X}^{L},\;C_{Y}^{L},\;C_{XY}^{L}$ and upper bounds $C_{X}^{U},\;C_{Y}^{U},\;C_{XY}^{U}$ are assumed unknown, therefore results in Section 2.4 are not applicable. In this set of experiments, we varied α in the range (0,1) to divide our original sample. We generated sample from an isotropic multivariate standard normal distribution (ρ=0) in all three scenarios (all features are independent). Therefore, the true GMI is zero and in all scenarios the GMI column, corresponding to the MST-GMI, is compared with zero. In each scenario we fixed dimension *d* and sample size *n* and varied α=0.2,0.5,0.8. The dimension and sample size in Scenarios 1,2, and 3 are d=6,8,10 and n=1000,1500,2000, respectively. In Table 1 the last column (α) stars the parameter α∈{0.2,0.5,0.8} with the minimum MSE and GMI $\left(I_{\alpha }\right)$ in each scenario. Table 1 shows that in these sets of experiments when α=0.5, the GMI estimator has less MSE (i.e., is more accurate) than when α=0.2 or α=0.8. This experimentally demonstrates that if we split our training data, the proposed Algorithm 1 performs better with α=0.5.

**Figure 5:**
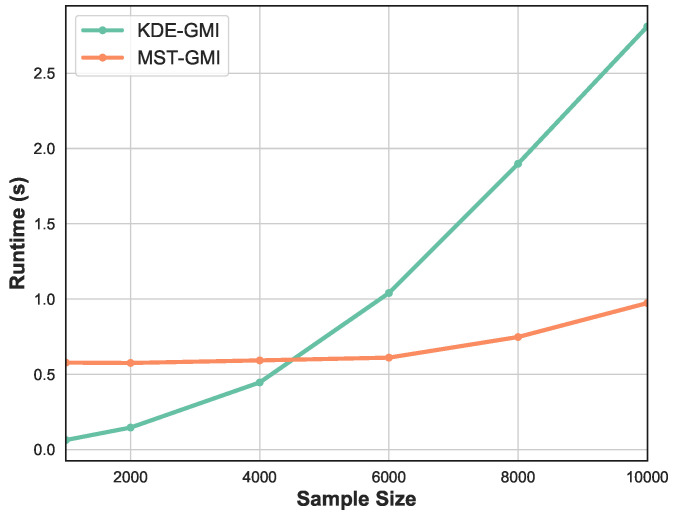
Runtime of KDE approach and proposed MST-based estimator of GMI vs sample size. The proposed GMI estimator achieves significant speedup, while for small sample size, the KDE method becomes overly fast. Please note that in this experiment the sample is generated from the Gaussian distribution in dimension d=2.

Finally, Figure 5 shows the runtime as a function of sample size *n*. We vary sample size in the range $\left[10^{3},10^{4}\right]$. Observe that for smaller number of samples the KDE-GMI method is slightly faster but as *n* becomes large we see significant relative speedup of the proposed MST-GMI method.

## 4. Conclusions

In this paper, we have proposed a new measure of mutual information, called Geometric MI (GMI), which is related to the Henze–Penrose divergence. The GMI can be viewed as dependency measure that is the limit of the Friedman–Rafsky test statistic, which depends on the MST over all data points. We established some properties of the GMI in terms of convexity/concavity, chain rule, and a type of data-processing inequality. A direct estimator of the GMI, called the MST-GMI, was introduced that uses random permutations of observed relationships between variables in the multivariate samples. An explicit form for the MSE convergence rate bound was derived that depends on a free parameter called the proportionality parameter. An asymptotically optimal form for this free parameter was given that minimizes the MSE convergence rate. Simulation studies were performed that illustrate and verify the theory.

## Figures and Tables

**Table 1:** Comparison between different scenarios of various dimensions and sample sizes in terms of parameter α. We applied the MST-GMI estimator to estimate the GMI ($I_{\alpha }$) with α=0.2,0.5,0.8. We varied dimension d=6,8,10 and sample size n=1000,1500,2000 in each scenario. We observe that for α={0.2,0.5,0.8}, the MST-GMI estimator provides lowest MSE when α=0.5 indicated by star (*).

Overview Table for Different *d*, *n*, and α					
Experiments	Dimension (d)	Sample Size (n)	GMI ($I_{\alpha }$)	MSE ($\times 10^{-4}$)	Parameter (α)
Scenario 1–1	6	1000	0.0229	12	0.2
Scenario 1–2	6	1000	0.0143	4.7944	0.5 *
Scenario 1–3	6	1000	0.0176	6.3867	0.8
Scenario 2–1	8	1500	0.0246	11	0.2
Scenario 2–2	8	1500	0.0074	1.6053	0.5 *
Scenario 2–3	8	1500	0.0137	5.3863	0.8
Scenario 3–1	10	2000	0.0074	2.3604	0.2
Scenario 3–2	10	2000	0.0029	0.54180	0.5 *
Scenario 3–3	10	2000	0.0262	11	0.8

## Appendix A

We organize the appendices as follows: Theorem 1 which establishes convexity/concavity is proved in Appendix A.1. Appendix A.2 and Appendix A.3 establish the inequality (9) and (10) for given p∈(0,1), respectively. In Appendix A.4, we first prove that the set ${\tilde{Z}}_{n^{\prime \prime }}$ which is randomly generated from original dependent data, contains asymptotically independent samples. Later by using the generated independent sample ${\tilde{Z}}_{n^{\prime \prime }}$ we show that for given α the FR estimator of the GMI given in Algorithm 1 tends to $I_{\alpha }$. Appendix A.5 dedicates proof of Theorem 4. The proportionality parameter (α) optimization strategy is presented in Appendix A.6.

### Appendix A.1. Theorem 1

**Proof.** The proof is similar to the result for standard (Shannon) mutual information. However, we require the following lemma, proven in analogous manner to the log-sum inequality:
**Lemma** **A1.** *For non-negative real numbers $\alpha_{1},\cdots ,\alpha_{n}$ and $\beta_{1},\cdots ,\beta_{n}$, given p∈(0,1), q=1−p,*

$$\sum_{i=1}^{n}\alpha_{i}\;{\left(p\left(\frac{\beta_{i}}{\alpha_{i}}\right)+q\right)}^{-1}\geq \sum_{i=1}^{n}\alpha_{i}{\left(p\left(\frac{\sum_{i=1}^{n}\beta_{i}}{\sum_{i=1}^{n}\alpha_{i}}\right)+q\right)}^{-1}.$$

Notice this follows by using the convex function $u\left(y\right)=y^{2}/\left(p+q\;y\right)$ for any p∈(0,1), q=1−p, and the Jensen inequality. Define the shorthand $\int_{x}$, $\int_{y}$, and $\int_{xy}$ for ∫dx, ∫dy and ∫∫dxdy, respectively. To prove part (i) of Theorem 1, we represent the LHS of (5) as:

$$\begin{array}{c} {\tilde{I}}_{p}\left(\lambda_{1}f_{Y|X}g_{X}+\lambda_{2}f_{Y|X}h_{X}\right)=1-\int_{xy}\left(\lambda_{1}f_{Y|X}g_{X}+\lambda_{2}f_{Y|X}h_{X}\right)\times \\ {\left[p\frac{\lambda_{1}f_{Y|X}g_{X}+\lambda_{2}f_{Y|X}h_{X}}{\left(\int_{x}\lambda_{1}f_{Y|X}g_{X}+\lambda_{2}f_{Y|X}h_{X}\right)\left(\int_{y}\lambda_{1}f_{Y|X}g_{X}+\lambda_{2}f_{Y|X}h_{X}\right)}+q\right]}^{-1}\\ =1-\int_{xy}\left(\lambda_{1}f_{Y|X}g_{X}+\lambda_{2}f_{Y|X}h_{X}\right){\left[p\frac{f_{Y|X}}{\left(\int_{x}\lambda_{1}f_{Y|X}g_{X}+\lambda_{2}f_{Y|X}h_{X}\right)}+q\right]}^{-1}. \end{array}$$

Furthermore, the RHS of (5) can be rewritten as

$$\begin{array}{c} \lambda_{1}{\tilde{I}}_{p}\left(f_{Y|X}g_{X}\right)+\lambda_{2}{\tilde{I}}_{p}\left(f_{Y|X}h_{X}\right)\\ =1-\int_{xy}\left(\lambda_{1}f_{Y|X}g_{X}{\left[p\frac{f_{Y|X}g_{X}}{\left(\int_{x}f_{Y|X}g_{X}\right)\left(\int_{y}f_{Y|X}g_{X}\right)}+q\right]}^{-1}+\lambda_{2}f_{Y|X}h_{X}{\left[p\frac{f_{Y|X}h_{X}}{\left(\int_{x}f_{Y|X}h_{X}\right)\left(\int_{y}f_{Y|X}h_{X}\right)}+q\right]}^{-1}\right)\\ \;=1-\int_{xy}\left(\lambda_{1}f_{Y|X}g_{X}{\left[p\frac{f_{Y|X}}{\int_{x}f_{Y|X}g_{X}}+q\right]}^{-1}+\lambda_{2}f_{Y|X}h_{X}{\left[p\frac{f_{Y|X}}{\int_{x}f_{Y|X}h_{X}}+q\right]}^{-1}\right). \end{array}$$

Thus, to prove LHS≥RHS, we use the inequality below:

$$\begin{array}{c} \left(\lambda_{1}f_{Y|X}g_{X}+\lambda_{2}f_{Y|X}h_{X}\right){\left[p\frac{f_{Y|X}}{\int_{x}\lambda_{1}f_{Y|X}g_{X}+\lambda_{2}f_{Y|X}h_{X}}+q\right]}^{-1}\\ \;\;\leq \left(\lambda_{1}f_{Y|X}g_{X}\right){\left[p\frac{\pi }{\int_{x}f_{Y|X}g_{X}}+q\right]}^{-1}+\left(\lambda_{2}f_{Y|X}h_{X}\right){\left[p\frac{\pi }{\int_{x}f_{Y|X}h_{X}}+q\right]}^{-1}. \end{array}$$

In Lemma A1, let

$$\begin{array}{c} \alpha_{1}=\frac{\lambda_{1}\left(\int_{x}f_{Y|X}g_{X}\right)\left(\lambda_{1}f_{Y|X}g_{X}+\lambda_{2}f_{Y|X}h_{X}\right)}{\int_{x}\lambda_{1}f_{Y|X}g_{X}+\lambda_{2}f_{Y|X}h_{X}},\\ \alpha_{2}=\frac{\lambda_{2}\left(\int_{x}f_{Y|X}h_{X}\right)\left(\lambda_{1}f_{Y|X}g_{X}+\lambda_{2}f_{Y|X}h_{X}\right)}{\int_{x}\lambda_{1}f_{Y|X}g_{X}+\lambda_{2}f_{Y|X}h_{X}}, \end{array}$$

and for i=1,2,

$$\beta_{i}=\frac{\lambda_{i}f_{Y|X}\left(\lambda_{1}f_{Y|X}g_{X}+\lambda_{2}f_{Y|X}h_{X}\right)}{\int_{x}\lambda_{1}f_{Y|X}g_{X}+\lambda_{2}f_{Y|X}h_{X}}.$$

Then the claimed assertion (i) is obtained. Part (ii) follows by convexity of $D_{p}$ and the following expression:

$$\begin{array}{c} {\tilde{I}}_{p}\left(\lambda_{1}g_{Y|X}f_{X}+\lambda_{2}h_{Y|X}f_{X}\right)\\ \;=D_{p}\left(\lambda_{1}f_{X}g_{Y|X}+\lambda_{2}f_{X}h_{Y|X}\;,\left(\int_{x}\lambda_{1}f_{X}g_{Y|X}+\lambda_{2}f_{X}h_{Y|X}\right)\left(\int_{y}\lambda_{1}\phi \pi_{1}+\lambda_{2}\phi \pi_{2}\right)\right)\\ \;=D_{p}(\lambda_{1}f_{X}g_{Y|X}+\lambda_{2}f_{X}h_{Y|X}\;,f_{X}\left(\int_{x}\lambda_{1}f_{X}g_{Y|X}+\lambda_{2}f_{X}h_{Y|X}\right)\\ \;=D_{p}\left(\lambda_{1}f_{X}g_{Y|X}+\lambda_{2}f_{X}h_{Y|X}\;,\lambda_{1}\left(\int_{x}f_{X}g_{Y|X}\right)\left(\int_{y}f_{X}g_{Y|X}\right)+\lambda_{2}\left(\int_{x}f_{X}h_{Y|X}\right)\left(\int_{y}f_{X}h_{Y|X}\right)\right). \end{array}$$

Therefore, the claim in (6) is proved. □

### Appendix A.2. Theorem 2

**Proof.** We start with (9). Given p∈(0,1) and q=1−p, we can easily check that for positive t>q, s>q, such that t,s≠1:

$$\left(t+s\right)\left[(ts)+q(1-t-s)\right]-p\left(ts\right)\geq 0.$$

This implies

$$p\left(\frac{t-q}{p}\right)\left(\frac{s-q}{p}\right)+q\geq \frac{t\;s}{t+s}.$$

By substituting

$$\frac{f_{X_{1}Y}\left(x_{1},y\right)}{f_{X_{1}}\left(x_{1}\right)\;f_{Y}\left(y\right)}=\frac{t-q}{p},\;\;\;\;\frac{f_{X_{2}Y|X_{1}}\left(x_{2},y|x_{1}\right)}{f_{X_{2}|X_{1}}\left(x_{2}|x_{1}\right)\;f_{Y|X_{1}}\left(y|x_{1}\right)}=\frac{s-q}{p},$$

we get

(A1) $$\begin{array}{c} {\left(p\;\frac{f_{X_{1}X_{2}Y}\left(x_{1},x_{2},y\right)}{f_{X_{1}X_{2}}\left(x_{1},x_{2}\right)\;f_{Y}\left(y\right)}+q\right)}^{-1}\leq {\left(p\;\frac{f_{X_{1}Y}\left(x_{1},y\right)}{f_{X_{1}}\left(x_{1}\right)\;f_{Y}\left(y\right)}+q\right)}^{-1}\\ \;\;+{\left(p\;\frac{f_{X_{2}Y|X_{1}}\left(x_{2},y|x_{1}\right)}{f_{X_{2}|X_{1}}\left(x_{2}|x_{1}\right)\;f_{Y|X_{1}}\left(y|x_{1}\right)}+q\right)}^{-1}. \end{array}$$

Consequently

(A2) $$\begin{array}{c} I_{p}\left(X_{1},X_{2};Y\right)\geq I_{p}\left(X_{1};Y\right)-E_{f}\left[{\left(p\;\frac{f_{X_{2}Y|X_{1}}\left(x_{2},y|x_{1}\right)}{f_{X_{2}|X_{1}}\left(x_{2}|x_{1}\right)\;f_{Y|X_{1}}\left(y|x_{1}\right)}+q\right)}^{-1}\right]. \end{array}$$

Here *f* is the joint PDF of random vector $\left(X_{1},X_{2},Y\right)$. From the conditional GMI definition in (7) the expectation term in (A2) is equivalent to $1-I_{p}\left(X_{2};Y|X_{1}\right)$. This completes the proof. □

### Appendix A.3. Proposition 1

**Proof.** Recall the Theorem 2, part (i). First from X→Y→Z we have $f_{XYZ}=f_{XY}f_{Z|Y}$ and then by applying the Jensen inequality,

(A3) $$\begin{array}{c} I_{p}\left(X;Y\right)=I_{p}\left(X;Y,Z\right)\;\;\;\;\;\;\;\mathrm{and}\\ I_{p}\left(X;Y,Z\right)\geq I_{p}\left(Z;X\right)-E\left[{\left(p\;\pi (X,Y,Z)+q\right)}^{-1}\right]\\ \;\;\;\geq I_{p}\left(Z;X\right)-{\left(p\;E\left[\pi (X,Y,Z)\right]+q\right)}^{-1}, \end{array}$$

where

$$\pi \left(x,y,z\right)=\frac{f_{YX|Z}\left(y,x|z\right)}{f_{Y|Z}\left(y|z\right)f_{X|Z}\left(x|z\right)}.$$

Now by Markovian property we can immediately simplify the RHS in (A3) to the RHS in (10). Furthermore, we can easily show that if X→Z→Y, we have $f_{XYZ}=f_{ZX}f_{Y|Z}$ and therefore $I_{p}\left(Z;X\right)=I_{p}\left(X;Y,Z\right)$. This together with (A3) proves that under both conditions X→Y→Z and X→Z→Y, the equality $I_{p}\left(X;Y\right)=I_{p}\left(Z;X\right)$ holds true. □

### Appendix A.4. Theorem 3

**Proof.** We first derive two required Lemmas A2 and A3 below:
**Lemma** **A2.** Consider random vector Z=(X,Y) with joint probability density function (pdf) $f_{XY}$. Let $Z_{n}=\left\{z_{1},\ldots ,z_{n}\right\}={\left\{\left(x_{i},y_{i}\right)\right\}}_{i=1}^{n}$ be a set of samples with pdf $f_{XY}$. Let $Z_{n^{\prime }}^{\prime }$ and $Z_{n^{\prime \prime }}^{\prime \prime }$ be two distinct subsets of $Z_{n}$ such that $n^{\prime }+n^{\prime \prime }=n$ and sample proportion is $\alpha =n^{\prime }/n$ and β=1−α. Next, let ${\tilde{Z}}_{n^{\prime \prime }}=\left\{{\tilde{z}}_{1},\ldots ,{\tilde{z}}_{n^{\prime \prime }}\right\}$ be a set of pairs such that ${\tilde{z}}_{k}=\left(x_{i_{k}},y_{j_{k}}\right)$, $k=1,\cdots ,n^{\prime \prime }$ are selected at random from $Z_{n^{\prime \prime }}^{\prime }$. Denote $\tilde{Z}=\left(\tilde{X},\tilde{Y}\right)$ as the random vector corresponding to samples in ${\tilde{Z}}_{n^{\prime \prime }}$. Then as n→∞ such that $n^{\prime \prime }$ also grows in a linked manner that β≠0 then the distribution of $\tilde{Z}$ convergences to $f_{X}\times f_{Y}$ i.e., random vectors $\tilde{X}$ and $\tilde{Y}$ become mutually independent.
**Proof.** Consider two subsets $A,\;B\subset R^{n}$, then we have

$$\begin{array}{c} P\left(\tilde{X}\in A,\tilde{Y}\in B\right)=E\left[I_{A}\left(\tilde{X}\right).\;I_{B}\left(\tilde{Y}\right)\right]=E\left[\sum_{i,j}I_{A}\left(X_{i}\right).\;I_{B}\left(Y_{j}\right).\;P\left(\left(\tilde{X},\tilde{Y}\right)=\left(X_{i},Y_{j}\right)|Z_{n}\right)\right]. \end{array}$$

Here $I_{A}$ stands for the indicator function. Please note that

$$P\left(\left(\tilde{X},\tilde{Y}\right)=\left(X_{i},Y_{j}\right)|Z_{n}\right)=\frac{1}{{n^{\prime \prime }}^{2}},$$

and $X_{i}$ and $Y_{j}$, i≠j are independent, therefore

$$\begin{array}{c} P\left(\tilde{X}\in A,\tilde{Y}\in B\right)=\frac{1}{{n^{\prime \prime }}^{2}}\sum_{i\neq j}P\left(X_{i}\in A\right)P\left(Y_{j}\in B\right)+\frac{1}{{n^{\prime \prime }}^{2}}\sum_{i=1}^{n}P\left(X_{i}\in A,Y_{i}\in B\right)\\ \;=P\left(X_{i}\in A\right)P\left(Y_{j}\in B\right)+\frac{1}{n^{\prime \prime }}\left\{P\left(X_{i}\in A,Y_{i}\in B\right)-P\left(X_{i}\in A\right)P\left(Y_{i}\in B\right)\right\}, \end{array}$$

this implies that

(A4) $$\begin{array}{c} |P\left(\tilde{X}\in A,\tilde{Y}\in B\right)-P\left(\tilde{X}\in A\right)P\left(\tilde{Y}\in B\right)|\leq \frac{1}{n^{\prime \prime }}\;\int \int \;|f_{XY}\left(x,y\right)-f_{X}\left(x\right)f_{Y}\left(y\right)|\;dx\;dy. \end{array}$$

On the other hand, we know that $n^{\prime \prime }=\beta \;n$, so we get

(A5) $$\begin{array}{c} |P\left(\tilde{X}\in A,\tilde{Y}\in B\right)-P\left(\tilde{X}\in A\right)P\left(\tilde{Y}\in B\right)|\leq \frac{1}{\beta \;n}\;\int \int \;|f_{XY}\left(x,y\right)-f_{X}\left(x\right)f_{Y}\left(y\right)|\;dx\;dy. \end{array}$$

From (A5), we observe that when β takes larger values the bound becomes tighter. So, if n→∞ such that $n^{\prime \prime }$ also becomes large enough in a linked manner so that β=constant then the RHS in (A5) tends to zero. This implies that $\tilde{X}$ and $\tilde{Y}$ become independent when n→∞. □ An immediate result of Lemma A2 is the following:
**Lemma** **A3.** *For given random vector $Z_{n}=\left(X_{n},Y_{n}\right)$ from joint density function $f_{XY}$ and with marginal density functions $f_{X}$ and $f_{Y}$ let ${\tilde{Z}}_{n^{\prime \prime }}=\left\{{\tilde{z}}_{1},\cdots ,{\tilde{z}}_{n^{\prime \prime }}\right\}$ be realization of random vector $\tilde{Z}$ as in Lemma A2 with parameter $\beta =n^{\prime \prime }/n$. Then for given points of ${\tilde{Z}}_{n^{\prime \prime }}$ at $\tilde{z}=\left(\tilde{x},\tilde{y}\right)$, we have*

(A6) $$|f_{\tilde{Z}}\left(\tilde{x},\tilde{y}\right)-f_{X}\left(\tilde{x}\right)f_{Y}\left(\tilde{y}\right)|=O\left(\frac{1}{\beta n}\right).$$

Now, we want to provide a proof of assertion (11). Consider two subsets $Z_{n^{\prime }}^{\prime }$ and ${\tilde{Z}}_{n^{\prime \prime }}$ as described in Section 2.2. Assume that the components of sample ${\tilde{Z}}_{n^{\prime \prime }}$ follow density function ${\tilde{f}}_{\tilde{X}\tilde{Y}}$. Therefore by owing to Lemmas A2 and A3, when n→∞ then ${\tilde{f}}_{\tilde{X}\tilde{Y}}\to f_{X}f_{Y}$. Let $M_{n^{\prime }}$ and $N_{n^{\prime \prime }}$ be Poisson variables with mean $n^{\prime }$ and $n^{\prime \prime }$ independent of one another and $\left\{Z_{i}^{\prime }\right\}$ and $\left\{{\tilde{Z}}_{j}\right\}$. Assume two Poisson processes $Z_{n^{\prime }}^{\prime }=\left\{Z_{1}^{\prime },\cdots ,Z_{M_{n^{\prime }}}^{\prime }\right\}$ and ${\tilde{Z}}_{n^{\prime \prime }}=\left\{{\tilde{Z}}_{1},\cdots ,{\tilde{Z}}_{N_{n^{\prime \prime }}}\right\}$, and denote the FR statistic $R_{n^{\prime },n^{\prime \prime }}^{\prime }$ on these processes. Following the arguments in [13,46] we shall prove the following:

$$\frac{E\left[R_{n^{\prime },n^{\prime \prime }}^{\prime }\right]}{n^{\prime }+n^{\prime \prime }}\to 2\alpha \beta \;\;\int \int \;\frac{f_{X,Y}\left(x,y\right)f_{X}\left(x\right)f_{Y}\left(y\right)}{\alpha f_{XY}\left(x,y\right)+\beta f_{X}\left(x\right)f_{Y}\left(y\right)}\;dx\;dy.$$

This follows due to $|R_{n^{\prime },n^{\prime \prime }}^{\prime }-R_{n^{\prime },n^{\prime \prime }}|\leq K_{d}\left(|M_{n^{\prime }}-n^{\prime }\left|+\right|N_{n^{\prime \prime }}-n^{\prime \prime }|\right)$, where $K_{d}$ is a constant defined in Lemma 1, [13] and $n^{\prime }+n^{\prime \prime }=n$. Thus, ${\left(n^{\prime }+n^{\prime \prime }\right)}^{-1}\;E|R_{n^{\prime },n^{\prime \prime }}^{\prime }-R_{n^{\prime },n^{\prime \prime }}|\to 0$ as n→∞. Let $W_{1}^{n^{\prime },n^{\prime \prime }},W_{2}^{n^{\prime },n^{\prime \prime }},\cdots$ be independent variables with common density

$$\phi_{n^{\prime },n^{\prime \prime }}\left(x,y\right)=\left(n^{\prime }f_{XY}\left(x,y\right)+n^{\prime \prime }{\tilde{f}}_{\tilde{X}\tilde{Y}}\left(x,y\right)\right)/\left(n^{\prime }+n^{\prime \prime }\right),$$

for $\left(x,y\right)\in R^{d}\times R^{d}$. Let $L_{n^{\prime },n^{\prime \prime }}$ be an independent Poisson variable with mean $n^{\prime }+n^{\prime \prime }$. Let $F_{n^{\prime },n^{\prime \prime }}^{\prime }=\left\{W_{1}^{n^{\prime },n^{\prime \prime }},\cdots ,W_{L_{n^{\prime },n^{\prime \prime }}}^{n^{\prime },n^{\prime \prime }}\right\}$ a non-homogeneous Poisson process of rate $n^{\prime }\;f_{XY}+n^{\prime \prime }\;{\tilde{f}}_{\tilde{X}\tilde{Y}}$. Assign mark 1 to a point in $F_{n^{\prime },n^{\prime \prime }}^{\prime }$ with probability

$$n^{\prime }\;f_{XY}\left(x,y\right)/\left(n^{\prime }\;f_{XY}\left(x,y\right)+n^{\prime \prime }\;{\tilde{f}}_{\tilde{X}\tilde{Y}}\left(x,y\right)\right),$$

and mark 2 otherwise. By the marking theorem [13,47], the FR test statistic ${\tilde{R}}_{n^{\prime },n^{\prime \prime }}$ has the same distribution as $R_{n^{\prime },n^{\prime \prime }}^{\prime }$. Given points of $F_{n^{\prime },n^{\prime \prime }}^{\prime }$ at $z^{\prime }=\left(x^{\prime },y^{\prime }\right)$ and $z^{\prime \prime }=\left(x^{\prime \prime },y^{\prime \prime }\right)$, the probability that they have different marks is given by (A7).

(A7) $$g_{n^{\prime },n^{\prime \prime }}\left(z^{\prime },z^{\prime \prime }\right)=\frac{n^{\prime }\;f_{XY}\left(x^{\prime },y^{\prime }\right)\;n^{\prime \prime }\;{\tilde{f}}_{\tilde{X},\tilde{Y}}\left(x^{\prime \prime },y^{\prime \prime }\right)+n^{\prime \prime }\;{\tilde{f}}_{\tilde{X},\tilde{Y}}\left(x^{\prime },y^{\prime }\right)\;n^{\prime }\;f_{XY}\left(x^{\prime \prime },y^{\prime \prime }\right)}{\left(n^{\prime }\;f_{XY}\left(x^{\prime },y^{\prime }\right)+n^{\prime \prime }\;{\tilde{f}}_{\tilde{X},\tilde{Y}}\left(x^{\prime },y^{\prime }\right)\right)\left(n^{\prime }\;f_{XY}\left(x^{\prime \prime },y^{\prime \prime }\right)+n^{\prime \prime }\;{\tilde{f}}_{\tilde{X},\tilde{Y}}\left(x^{\prime \prime },y^{\prime \prime }\right)\right)},$$

define

(A8) $$g\left(z^{\prime },z^{\prime \prime }\right)=\frac{\alpha \beta \left(f_{XY}\left(x^{\prime },y^{\prime }\right)f_{X}\left(x^{\prime \prime }\right)f_{Y}\left(y^{\prime \prime }\right)+f_{X}\left(x^{\prime }\right)f_{Y}\left(y^{\prime }\right)f_{XY}\left(x^{\prime \prime },y^{\prime \prime }\right)\right)}{\left(\alpha \;f_{XY}\left(x^{\prime \prime },y^{\prime \prime }\right)+\beta f_{X}\left(x^{\prime \prime }\right)f_{Y}\left(y^{\prime \prime }\right)\right)\left(\alpha f_{XY}\left(x^{\prime },y^{\prime }\right)+\beta f_{X}\left(x^{\prime }\right)f_{Y}\left(y^{\prime }\right)\right)},$$

then

(A9) $$\begin{array}{c} E\left[{\tilde{R}}_{n^{\prime },n^{\prime \prime }}^{\prime }|F_{n^{\prime },n^{\prime \prime }}^{\prime }\right]=\underset{i<j\leq L_{n^{\prime },n^{\prime \prime }}}{\sum \sum }g_{n}\left(W_{i}^{n^{\prime },n^{\prime \prime }},W_{j}^{n^{\prime },n^{\prime \prime }}\right)I_{F_{n^{\prime },n^{\prime \prime }}^{\prime }}\left(W_{i}^{n^{\prime },n^{\prime \prime }},W_{j}^{n^{\prime },n^{\prime \prime }}\right). \end{array}$$

Now recall (A8). We observe that $g_{n^{\prime },n^{\prime \prime }}\left(z^{\prime },z^{\prime \prime }\right)\to g\left(z^{\prime },z^{\prime \prime }\right)$. Going back to (A9), we can write

(A10) $$\begin{array}{c} E\left[{\tilde{R}}_{n^{\prime },n^{\prime \prime }}^{\prime }\right]=\underset{i<j\leq L_{n^{\prime },n^{\prime \prime }}}{\sum \sum }g_{n^{\prime },n^{\prime \prime }}\left(W_{i}^{n^{\prime },n^{\prime \prime }},W_{j}^{n^{\prime },n^{\prime \prime }}\right)I_{F_{n^{\prime },n^{\prime \prime }}^{\prime }}\left(W_{i}^{n^{\prime },n^{\prime \prime }},W_{j}^{n^{\prime },n^{\prime \prime }}\right)+o\left(n^{\prime }+n^{\prime \prime }\right). \end{array}$$

For fixed $n^{\prime }$, $n^{\prime \prime }$ consider the collection:

$$F_{n^{\prime },n^{\prime \prime }}=\left\{W_{1}^{n^{\prime },n^{\prime \prime }},\cdots ,W_{n^{\prime },n^{\prime \prime }}^{n^{\prime }+n^{\prime \prime }}\right\}.$$

By the fact that $E\left[M_{n^{\prime }}+N_{n^{\prime \prime }}-\left(n^{\prime }+n^{\prime \prime }\right)\right]=o\left(n^{\prime }+n^{\prime \prime }\right)$, we have

(A11) $$\begin{array}{c} E\left[{\tilde{R}}_{n^{\prime },n^{\prime \prime }}^{\prime }\right]=\underset{i<j\leq n^{\prime }+n^{\prime \prime }}{\sum \sum }g_{n^{\prime },n^{\prime \prime }}\left(W_{i}^{n^{\prime },n^{\prime \prime }},W_{j}^{n^{\prime },n^{\prime \prime }}\right)I_{F_{n^{\prime },n^{\prime \prime }}}\left(W_{i}^{n^{\prime }},W_{j}^{n^{\prime \prime }}\right)+o\left(n^{\prime }+n^{\prime \prime }\right). \end{array}$$

Introduce

$$\phi \left(x,y\right)=\alpha f_{XY}\left(x,y\right)+\beta f_{X}\left(x\right)f_{Y}\left(y\right).$$

Then $\phi_{n^{\prime },n^{\prime \prime }}\left(x,y\right)\to \phi \left(x,y\right)$ uniformly as $n^{\prime }/n\to \alpha$ and $n^{\prime \prime }/n\to \beta$. Thus, using Proposition 1 in [13], we get

(A12) $$\begin{array}{c} \frac{E\left[{\tilde{R}}_{n^{\prime },n^{\prime \prime }}^{\prime }\right]}{n}\to \int g\left(z,z\right)\phi \left(z\right)dz=\;\int \int \;\frac{2\alpha \beta f_{XY}\left(x,y\right)f_{X}\left(x\right)f_{Y}\left(y\right)}{\alpha f_{XY}\left(x,y\right)+\beta f_{X}\left(x\right)f_{Y}\left(y\right)}\;dx\;dy. \end{array}$$

 □

### Appendix A.5. Theorem 4

**Proof.** We begin by providing a family of bias rate bounds for the FR test statistic $R_{n^{\prime },n^{\prime \prime }}$ in terms of a parameter *l*. Assume $f_{XY}$, $f_{X}$, and $f_{Y}$ are in $\Sigma_{d}\left(\eta ,K\right)$. Then by plugging the optimal *l*, we prove the bias rate bound given in (13).
**Theorem** **A1.** *Let $R_{n^{\prime },n^{\prime \prime }}:=R\left(Z_{n^{\prime }},Z_{n^{\prime \prime }}\right)$ be the FR test statistic. Then a bound on the bias rate of the $R_{n^{\prime },n^{\prime \prime }}$ estimator for 0<η≤1, d≥2 is given by*

(A13) $$\begin{array}{c} \left|\frac{E\left[R_{n^{\prime },n^{\prime \prime }}\right]}{n}-2\alpha \beta \;\int \int \;\frac{f_{XY}\left(x,y\right)f_{X}\left(x\right)f_{Y}\left(y\right)}{\alpha f_{XY}\left(x,y\right)+\beta f_{X}\left(x\right)f_{Y}\left(y\right)}\;dx\right|\\ \leq O\left(l^{d}{\left(n\right)}^{-\eta /d}\right)+O\left(l^{-d\eta }\right)+O\left(l^{d}\beta^{-1}n^{-1}\right)+O\left(c_{d}n^{-1}\right), \end{array}$$

*where 0<η≤1 is the Hölder smoothness parameter and $c_{d}$ is the largest possible degree of any vertex of MST.* *Set*

$$\begin{array}{c} \alpha_{i}=\alpha na_{i}l^{d}\;\left(1-a_{i}l^{-d}\right)+{\left(\alpha n\right)}^{2}a_{i}^{2},\\ \beta_{i}=\beta nb_{i}l^{d}\;\left(1-b_{i}l^{-d}\right)+{\left(\beta n\right)}^{2}b_{i}^{2}. \end{array}$$

*and*

(A14) $$A_{f,n}^{\beta ,\alpha }\left(x,y\right)=\frac{2f_{XY}\left(x,y\right)\left(f_{X}\left(x\right)f_{Y}\left(y\right)+\delta_{f}/\left(\beta n\right)\right)\left(f_{XY}\left(x,y\right)\sqrt{\alpha }+(f_{X}\left(x\right)f_{Y}\left(y\right)+\delta_{f}/\left(\beta n\right))\sqrt{\beta }\right)}{a_{i}^{2}l^{-d}{\left(\alpha f_{XY}\left(x,y\right)+\beta \left(f_{X}\left(x\right)f_{Y}\left(y\right)+\delta_{f}/\left(\beta n\right)\right)\right)}^{2}},$$

*where*

(A15) $$\delta_{f}=\;\int \int \;|f_{XY}\left(x,y\right)-f_{X}\left(x\right)f_{Y}\left(y\right)|\;dxdy,$$

*A more explicit form for the bound on the RHS is given below:*

(A16) $$\begin{array}{c} \Delta \left(\alpha ,f_{XY},f_{X}f_{Y}\right):=c_{2}l^{d}{\left(n\right)}^{-1}+c_{d}2^{d}{\left(n\right)}^{-1}+O\left(l^{d}{\left(n\right)}^{-\eta /d}\right)+O\left(l^{d}{\left(n\right)}^{-1/2}\right)\\ +O\left(c_{d}{\left(n\right)}^{-1/2}\right)+2c_{1}l^{d-1}{\left(n\right)}^{(1/d)-1}+\delta_{f}\left({\left(\beta n\right)}^{-1}\right)\;\int \int \;\frac{2\alpha \beta f_{XY}\left(x,y\right)}{\alpha f_{XY}\left(x,y\right)+\beta f_{X}\left(x\right)f_{Y}\left(y\right)}\;dxdy\\ +{\left(n\right)}^{-1}\sum_{i=1}^{M}2\;\int \int \;f_{XY}\left(x,y\right)\left(f_{X}\left(x\right)f_{Y}\left(y\right)+\delta_{f}/\left(\beta n\right)\right)(\alpha_{i}\beta_{i}(\alpha na_{i}l^{-d}f_{XY}^{2}\left(x,y\right)\\ \;+\beta nb_{i}l^{-d}{\left(f_{X}\left(x\right)f_{Y}\left(y\right)+\delta_{f}/\left(\beta n\right)\right)}^{2}){)}^{1/2}/{\left(\alpha na_{i}f_{XY}\left(x,y\right)+\beta nb_{i}f_{X}\left(x\right)f_{Y}\left(y\right)\right)}^{2}\;dxdy\\ \;+{\left(n\right)}^{-1}\sum_{i=1}^{M}O\left(l\right)\;\int \int \;l^{d}{\left(a_{i}\right)}^{-1}\frac{2f_{XY}\left(x,y\right)\left(f_{X}\left(x\right)f_{Y}\left(y\right)+\delta_{f}/\left(\beta n\right)\right)}{\alpha f_{XY}\left(x,y\right)+\beta f_{X}\left(x\right)f_{Y}\left(y\right)}\;dxdy+O\left(l^{-d\eta }\right)\\ \;+{\left(n\right)}^{-3/2}\sum_{i=1}^{M}O\left(l\right)\;\int \int \;l^{-d/2}\sqrt{b_{i}}\;A_{f,n}^{\beta ,\alpha }\left(x,y\right)\;dxdy. \end{array}$$

**Proof.** Consider two Poisson variables $M_{n^{\prime }}$ and $N_{n^{\prime \prime }}$ with mean $n^{\prime }$ and $n^{\prime \prime }$ respectively and independent of one another and $\left\{Z_{i}^{\prime }\right\}$ and $\left\{{\tilde{Z}}_{j}\right\}$. Let $Z_{n^{\prime }}^{\prime }$ and ${\tilde{Z}}_{n^{\prime \prime }}$ be the Poisson processes $\left\{Z_{1}^{\prime },\cdots ,Z_{M_{n^{\prime }}}^{\prime }\right\}$ and $\left\{{\tilde{Z}}_{1},\cdots ,{\tilde{Z}}_{N_{n^{\prime \prime }}}\right\}$. Likewise Appendix A.4, set $R_{n^{\prime },n^{\prime \prime }}^{\prime }=R\left(Z_{n^{\prime }}^{\prime },{\tilde{Z}}_{n^{\prime \prime }}\right)$. Applying Lemma 1, and (12) in [13], we can write

(A17) $$|R_{n^{\prime },n^{\prime \prime }}^{\prime }-R_{n^{\prime },n^{\prime \prime }}|\leq c_{d}\left(\right|M_{n^{\prime }}-n^{\prime }\left|+\right|N_{n^{\prime \prime }}-n^{\prime \prime }\left|\right).$$

Here $c_{d}$ denotes the largest possible degree of any vertex of the MST in $R^{d}$. Following the arguments in [46], we have $E\left[|M_{n^{\prime }}-n^{\prime }|\right]=O\left(\;{n^{\prime }}^{1/2}\right)$ and $E\left[|N_{n^{\prime \prime }}-n^{\prime \prime }|\right]=O\left(\;{n^{\prime \prime }}^{1/2}\right)$. Hence

(A18) $$\frac{E\left[R_{n^{\prime },n^{\prime \prime }}\right]}{n^{\prime }+n^{\prime \prime }}=\frac{E\left[R_{n^{\prime },n^{\prime \prime }}^{\prime }\right]}{n^{\prime }+n^{\prime \prime }}+O\left(c_{d}{\left(n^{\prime }+n^{\prime \prime }\right)}^{-1/2}\right).$$

Next let $n_{i}^{\prime }$ and $n_{i}^{\prime \prime }$ be independent binomial random variables with marginal densities $B(n^{\prime },a_{i}l^{-d})$ and $B(n^{\prime \prime },b_{i}l^{-d})$ such that $a_{i},b_{i}$ are non-negative constants $a_{i}\leq b_{i}$ and $\sum_{i=1}^{l^{d}}a_{i}l^{-d}=\sum_{i=1}^{l^{d}}b_{i}l^{-d}=1$. Therefore, using the subadditivity property in Lemma 2.2, [46], we can write

(A19) $$\begin{array}{c} E\left[R_{n^{\prime },n^{\prime \prime }}^{\prime }\right]\leq \sum_{i=1}^{M}E\left[E\left[R_{n_{i}^{\prime },n_{i}^{\prime \prime }}^{\prime }|n_{i}^{\prime },n_{i}^{\prime \prime }\right]\right]+2\;c_{1}\;l^{d-1}{\left(n^{\prime }+n^{\prime \prime }\right)}^{1/d}, \end{array}$$

where $M=l^{d}$, and η>0 is the Hölder smoothness parameter. Furthermore, for given $n_{i}^{\prime }$, $n_{i}^{\prime \prime }$, let $W_{1}^{n_{i}^{\prime },n_{i}^{\prime \prime }},W_{2}^{n_{i}^{\prime },n_{i}^{\prime \prime }},\cdots$ be independent variables with common densities for $\left(x,y\right)\in R^{d}\times R^{d}$:

$$g_{n_{i}^{\prime },n_{i}^{\prime \prime }}\left(x,y\right)=\left(n_{i}^{\prime }f_{XY}\left(x,y\right)+n_{i}^{\prime \prime }{\tilde{f}}_{\tilde{X}\tilde{Y}}\left(x,y\right)\right)/\left(n_{i}^{\prime }+n_{i}^{\prime \prime }\right).$$

Denote $L_{n_{i}^{\prime },n_{i}^{\prime \prime }}$ be an independent Poisson variable with mean $n_{i}^{\prime }+n_{i}^{\prime \prime }$ and $F_{n_{i}^{\prime },n_{i}^{\prime \prime }}^{\prime }=\left\{W_{1}^{n_{i}^{\prime },n_{i}^{\prime \prime }},\cdots ,W_{L_{n_{i}^{\prime }.n_{i}^{\prime \prime }}}^{n_{i}^{\prime },n_{i}^{\prime \prime }}\right\}$ a non-homogeneous Poisson of rate $n_{i}^{\prime }f_{XY}+n_{i}^{\prime \prime }{\tilde{f}}_{\tilde{X}\tilde{Y}}$. Let $F_{n_{i}^{\prime },n_{i}^{\prime \prime }}$ be the non-Poisson point process $\left\{W_{1}^{n_{i}^{\prime },n_{i}^{\prime \prime }},\cdots W_{n_{i}^{\prime }+n_{i}^{\prime \prime }}^{n_{i}^{\prime },n_{i}^{\prime \prime }}\right\}$. Assign a mark from the set {1,2} to each point of $F_{n_{i}^{\prime },n_{i}^{\prime \prime }}^{\prime }$. Let ${\tilde{Z}}_{n_{i}^{\prime }}^{\prime }$ be the sets of points marked 1 with each probability $n_{i}^{\prime }f_{XY}\left(x,y\right)/\left(n_{i}^{\prime }f_{XY}\left(x,y\right)+n_{i}^{\prime \prime }{\tilde{f}}_{\tilde{X}\tilde{Y}}\left(x,y\right)\right)$ and let ${\tilde{Z}}_{n_{i}^{\prime \prime }}^{\prime \prime }$ be the set points with mark 2. Please note that owing to the marking theorem [47], ${\tilde{Z}}_{n_{i}^{\prime }}^{\prime }$ and ${\tilde{Z}}_{n_{i}^{\prime \prime }}^{\prime \prime }$ are independent Poisson processes with the same distribution as $Z_{n_{i}^{\prime }}^{\prime }$ and ${\tilde{Z}}_{n_{i}^{\prime \prime }}$, respectively. Considering ${\tilde{R}}_{n_{i}^{\prime },n_{i}^{\prime \prime }}^{\prime }$ as FR test statistic on nodes in ${\tilde{Z}}_{n_{i}^{\prime }}^{\prime }\cup {\tilde{Z}}_{n_{i}^{\prime \prime }}^{\prime \prime }$, we have

$$E\left[R_{n_{i}^{\prime },n_{i}^{\prime \prime }}^{\prime }|n_{i}^{\prime },n_{i}^{\prime \prime }\right]=E\left[{\tilde{R}}_{n_{i}^{\prime },n_{i}^{\prime \prime }}^{\prime }|n_{i}^{\prime },n_{i}^{\prime \prime }\right].$$

By the fact that $E\left[|M_{n^{\prime }}+N_{n^{\prime \prime }}-n^{\prime }-n^{\prime \prime }|\right]=O\left({\left(n^{\prime }+n^{\prime \prime }\right)}^{1/2}\right)$, we have

$$\begin{array}{c} E\left[{\tilde{R}}_{n_{i}^{\prime },n_{i}^{\prime \prime }}^{\prime }|n_{i}^{\prime },n_{i}^{\prime \prime }\right]=E\left[E\left[{\tilde{R}}_{n_{i}^{\prime },n_{i}^{\prime \prime }}^{\prime }|F_{n_{i}^{\prime },n_{i}^{\prime \prime }}^{\prime }\right]\right]\\ \;=E\left[\underset{s<j<n_{i}^{\prime }+n_{i}^{\prime \prime }}{\sum \sum }P_{n_{i}^{\prime },n_{i}^{\prime \prime }}\left(W_{s}^{n_{i}^{\prime },n_{i}^{\prime \prime }},W_{j}^{n_{i}^{\prime },n_{i}^{\prime \prime }}\right)\;1\left\{\left(W_{s}^{n_{i}^{\prime },n_{i}^{\prime \prime }},W_{j}^{n_{i}^{\prime },n_{i}^{\prime \prime }}\right)\in F_{n_{i}^{\prime },n_{i}^{\prime \prime }}\right\}\right]+O\left({\left(n_{i}^{\prime }+n_{i}^{\prime \prime }\right)}^{1/2})\right). \end{array}$$

Here $z^{\prime }=\left(x^{\prime },y^{\prime }\right)$, $z^{\prime \prime }=\left(x^{\prime \prime },y^{\prime \prime }\right)$, and $P_{n_{i}^{\prime },n_{i}^{\prime \prime }}\left(z^{\prime },z^{\prime \prime }\right)$ is given in below:

$$\begin{array}{c} P_{n_{i}^{\prime },n_{i}^{\prime \prime }}\left(z^{\prime },z^{\prime \prime }\right):=P_{r}\left\{\mathrm{mark}\;z^{\prime }\neq \mathrm{mark}\;z^{\prime \prime },\left(z^{\prime },z^{\prime \prime }\right)\in F_{n_{i}^{\prime },n_{i}^{\prime \prime }}\right\}\\ =\frac{n_{i}^{\prime }f_{XY}\left(x^{\prime },y^{\prime }\right)n_{i}^{\prime \prime }{\tilde{f}}_{\tilde{X}\tilde{Y}}\left(x^{\prime \prime },y^{\prime \prime }\right)+n_{i}^{\prime }{\tilde{f}}_{\tilde{X}\tilde{Y}}\left(x^{\prime },y^{\prime }\right)n_{i}^{\prime \prime }f_{X,Y}\left(x^{\prime \prime },y^{\prime \prime }\right)}{\left(n_{i}^{\prime \prime }f_{XY}\left(x^{\prime },y^{\prime }\right)+n_{i}^{\prime \prime }{\tilde{f}}_{\tilde{X}\tilde{Y}}\left(x^{\prime },y^{\prime }\right)\right)\left(n_{1}^{\prime }f_{XY}\left(x^{\prime \prime },y^{\prime \prime }\right)+n_{i}^{\prime \prime }{\tilde{f}}_{\tilde{X}\tilde{Y}}\left(x^{\prime \prime },y^{\prime \prime }\right)\right)}. \end{array}$$

Next set

$$\begin{array}{c} \alpha_{i}=n^{\prime }a_{i}l^{d}\;\left(1-a_{i}l^{-d}\right)+{n^{\prime }}^{2}a_{i}^{2},\;\;\;\beta_{i}=n^{\prime \prime }b_{i}l^{d}\;\left(1-b_{i}l^{-d}\right)+{n^{\prime \prime }}^{2}b_{i}^{2}. \end{array}$$

By owing to the Lemma B.6 in [46] and applying analogous arguments, we can rewrite the expression in (A20):

(A20) $$\begin{array}{c} E\left[{R^{\prime }}_{n^{\prime },n^{\prime \prime }}\right]\leq \sum_{i=1}^{M}\;a_{i}b_{i}l^{-d}\;\int \int \;\frac{2\;n^{\prime }n^{\prime \prime }f_{XY}\left(x,y\right)f_{\tilde{X}\tilde{Y}}\left(x,y\right)}{n^{\prime }a_{i}f_{X,Y}\left(x,y\right)+n^{\prime \prime }b_{i}f_{\tilde{X}\tilde{Y}}\left(x,y\right)}\;dx\;dy+2c_{1}\;l^{d-1}{\left(n^{\prime }+n^{\prime \prime }\right)}^{1/d}\\ \;+\sum_{i=1}^{M}2\;\int \int \;\frac{f_{XY}\left(x,y\right)f_{\tilde{X}\tilde{Y}}\left(x,y\right){\left(\alpha_{i}\beta_{i}\left(n^{\prime }a_{i}l^{-d}f_{XY}^{2}\left(x,y\right)+n^{\prime \prime }b_{i}l^{-d}f_{\tilde{X}\tilde{Y}}^{2}\left(x,y\right)\right)\right)}^{1/2}}{{\left(n^{\prime }a_{i}f_{XY}\left(x,y\right)+n^{\prime \prime }b_{i}f_{\tilde{X}\tilde{Y}}\left(x,y\right)\right)}^{2}}\;dxdy\\ \;+\sum_{i=1}^{M}E_{n_{i}^{\prime },n_{i}^{\prime \prime }}\left[\left(n_{i}^{\prime }+n_{i}^{\prime \prime }\right)\;\varsigma_{\eta }\left(l,n_{i}^{\prime },n_{i}^{\prime \prime }\right)\right]+O\left(l^{d}{\left(n^{\prime }+n^{\prime \prime }\right)}^{1-\eta /d}\right)+O\left(l^{d}{\left(n^{\prime }+n^{\prime \prime }\right)}^{1/2}\right), \end{array}$$

where

$$\varsigma_{\eta }\left(l,n_{i}^{\prime },n_{i}^{\prime \prime }\right)=\left(O\left(\frac{l}{n_{i}^{\prime }+n_{i}^{\prime \prime }}\right)-\frac{2\;l^{d}}{n_{i}^{\prime }+n_{i}^{\prime \prime }}\right)\int g_{n_{i}^{\prime },n_{i}^{\prime \prime }}\left(z^{\prime }\right)P_{n_{i}^{\prime },n_{i}^{\prime \prime }}\left(z^{\prime },z^{\prime }\right)\;dz^{\prime }+O\left(l^{-d\eta }\right).$$

Going back to Lemma A3, we know that

$$f_{\tilde{X}\tilde{Y}}\left(x,y\right)=f_{X}\left(x\right)f_{Y}\left(y\right)+O\left(\frac{1}{\beta n}\right).$$

Therefore, the first term on the RHS of (A20) is less and equal to

$$\begin{array}{c} \sum_{i=1}^{M}\;a_{i}b_{i}l^{-d}\;\int \int \;\frac{2\;n^{\prime }n^{\prime \prime }f_{XY}\left(x,y\right)f_{X}\left(x\right)f_{Y}\left(y\right)}{n^{\prime }a_{i}f_{X,Y}\left(x,y\right)+n^{\prime \prime }b_{i}f_{X}\left(x\right)f_{Y}\left(y\right)}\;dx\;dy\\ +\left(\frac{\delta_{f}}{\beta n}\right)\sum_{i=1}^{M}\;a_{i}b_{i}l^{-d}\;\int \int \;\frac{2\;n^{\prime }n^{\prime \prime }f_{XY}\left(x,y\right)}{n^{\prime }a_{i}f_{XY}\left(x,y\right)+n^{\prime \prime }b_{i}f_{X}\left(x\right)f_{Y}\left(y\right)}\;dx\;dy, \end{array}$$

and the second term is less and equal to

$$\begin{array}{c} \sum_{i=1}^{M}2\;\int \int \;f_{XY}\left(x,y\right)\left(f_{X}\left(x\right)f_{Y}\left(y\right)+\delta_{f}/\left(\beta n\right)\right)(\alpha_{i}\beta_{i}(n^{\prime }a_{i}l^{-d}f_{XY}^{2}\left(x,y\right)+n^{\prime \prime }b_{i}l^{-d}(f_{X}\left(x\right)f_{Y}\left(y\right)\\ \;\;+\delta_{f}/\left(\beta n\right){)}^{2}){)}^{1/2}/{\left(n^{\prime }a_{i}f_{XY}\left(x,y\right)+n^{\prime \prime }b_{i}f_{X}\left(x\right)f_{Y}\left(y\right)\right)}^{2}\;dxdy, \end{array}$$

where

$$\delta_{f}=\;\int \int \;|f_{XY}\left(x,y\right)-f_{X}\left(x\right)f_{Y}\left(y\right)|\;dxdy.$$

Recall the definition of the dual MST and FR statistic denoted by $R_{n^{\prime },n^{\prime \prime }}^{*}$ following [46]:
**Definition** **A1.** (Dual MST, ${\mathrm{MST}}^{*}$ and dual FR statistic $R_{m,n}^{*}$) *Let $F_{i}$ be the set of corner points associated with a particular subsection $Q_{i}$, $1\leq i\leq l^{d}$ of ${\left[0,1\right]}^{d}$. Define the dual ${\mathrm{MST}}^{*}\left(X_{m}\cup Y_{n}\cap Q_{i}\right)$ as the boundary MST graph in partition $Q_{i}$ [48], which contains $X_{m}$ and $Y_{n}$ points falling inside partition cell $Q_{i}$ and those corner points in $F_{i}$ which minimize total MST length. Please note that it is allowed to connect the MSTs in $Q_{i}$ and $Q_{j}$ through points strictly contained in $Q_{i}$ and $Q_{j}$ and therefore corner points. Thus, the dual MST can connect the points in $Q_{i}\cup Q_{j}$ by direct edges to pair to another point in $Q_{i}\cup Q_{j}$ or by passing through the corner the corner points which are all connected in order to minimize the total weights. To clarify, assume that there are two points in $Q_{i}\cup Q_{j}$, then the dual MST consists of the two edges connecting these points to the corner if they are closer to a corner point otherwise the dual MST connects them to each other.* Furthermore, $R_{m,n}^{*}\left(X_{m},Y_{n}\cap Q_{i}\right)$ is defined as the number of edges in the ${\mathrm{MST}}^{*}$ graph connecting nodes from different samples and number of edges connecting to the corner points. Please note that the edges connected to the corner nodes (regardless of the type of points) are always counted in the dual FR test statistic $R_{m,n}^{*}$. Similarly, consider the Poisson processes samples and the FR test statistic over these samples, denoted by ${R^{\prime }}_{n^{\prime },n^{\prime \prime }}^{*}$. By superadditivity of the dual $R_{n^{\prime },n^{\prime \prime }}^{*}$ [46], we have

(A21) $$\begin{array}{c} E\left[R_{n^{\prime },n^{\prime \prime }}^{\prime *}\right]\geq \sum_{i=1}^{M}a_{i}\;l^{-d}\;\int \int \;\frac{2n^{\prime }n^{\prime \prime }f_{XY}\left(x,y\right)\left(f_{X}\left(x\right)f_{Y}\left(y\right)-\delta_{f}/\left(\beta n\right)\right)}{n^{\prime }f_{XY}\left(x,y\right)+n^{\prime \prime }\left(f_{X}\left(x\right)f_{Y}\left(y\right)-\delta_{f}/\left(\beta n\right)\right)}\;dxdy\\ \;-\sum_{i=1}^{M}E_{n_{i}^{\prime },n_{i}^{\prime \prime }}\left[\left(n_{i}^{\prime }+n_{i}^{\prime \prime }\right)\;\varsigma_{\eta }\left(l,n_{i}^{\prime },n_{i}^{\prime \prime }\right)\right]-O\left(l^{d}{\left(n^{\prime }+n^{\prime \prime }\right)}^{1-\eta /d}\right)-O\left(l^{d}{\left(n^{\prime }+n^{\prime \prime }\right)}^{1/2}\right)-c_{2}\;l^{d}. \end{array}$$

The first term of RHS in (A21) is greater or equal to

$$\begin{array}{c} \;\int \int \;\frac{2n^{\prime }n^{\prime \prime }f_{XY}\left(x,y\right)f_{X}\left(x\right)f_{Y}\left(y\right)}{n^{\prime }f_{XY}\left(x,y\right)+n^{\prime \prime }f_{X}\left(x\right)f_{Y}\left(y\right)}\;dxdy-\frac{\delta_{f}}{\beta n}\;\int \int \;\frac{2n^{\prime }n^{\prime \prime }f_{XY}\left(x,y\right)}{n^{\prime }f_{XY}\left(x,y\right)+n^{\prime \prime }f_{X}\left(x\right)f_{Y}\left(y\right)}\;dxdy. \end{array}$$

Furthermore,

$$\frac{E\left[R_{n^{\prime },n^{\prime \prime }}^{\prime }\right]}{n}+\frac{c_{d}2^{d}}{n}\geq \frac{E\left[R_{n^{\prime },n^{\prime \prime }}^{\prime *}\right]}{n},$$

where $c_{d}$ is the largest possible degree of any vertex of the MST in $R^{d}$, as before. Consequently, we have

(A22) $$\begin{array}{c} |\frac{E\left[R_{n^{\prime },n^{\prime \prime }}^{\prime }\right]}{n}-\;\int \int \;\frac{2\alpha \beta f_{XY}\left(x,y\right)f_{X}\left(x\right)f_{Y}\left(y\right)}{\alpha f_{XY}\left(x,y\right)+\beta f_{X}\left(x\right)f_{Y}\left(y\right)}\;dxdy|\leq B\left(\alpha ,f_{XY},f_{X}f_{Y}\right), \end{array}$$

where B is defined in (A16) and $A_{f,n}^{\beta ,\alpha }\left(x,y\right)$ has been introduced in (A14). The last line in (A22) follows from the fact that

$$\begin{array}{c} \sum_{i=1}^{M}E_{n_{i}^{\prime },n_{i}^{\prime \prime }}\left[\left(n_{i}^{\prime }+n_{i}^{\prime \prime }\right)\;\varsigma_{\eta }\left(l,n_{i}^{\prime },n_{i}^{\prime \prime }\right)\right]\leq \sum_{i=1}^{M}O\left(l\right)\;\int \int \;l^{-d/2}\sqrt{b_{i}}\;A_{f,n}^{\beta ,n^{\prime }/n}\left(x,y\right)dxdy\\ \;+\sum_{i=1}^{M}O\left(l\right)\;\int \int \;l^{d}{\left(a_{i}\right)}^{-1}\frac{2f_{XY}\left(x,y\right)\left(f_{X}\left(x\right)f_{Y}\left(y\right)+O\left(\delta_{f}/\left(\beta n\right)\right)\right)}{n^{\prime }f_{XY}\left(x,y\right)+n^{\prime \prime }f_{X}\left(x\right)f_{Y}\left(y\right)}\;dxdy. \end{array}$$

Here $A_{f,n}^{\beta ,n^{\prime }/n}\left(x,y\right)$ is given as (A14) by substituting $n^{\prime }/n$ in α such that β=1−α. Hence, the proof of Theorem A1 is completed. □ Going back to the proof of (13), without loss of generality assume that $\left(n\right)l^{-d}>1$, for d≥2 and 0<η≤1. We select *l* as a function of *n* and β to be the sequence increasing in *n* which minimizes the maximum of these rates:

(A23) $$l\left(n,\beta \right)=arg\;\min_{l}\max\left\{l^{d}{\left(n\right)}^{-\eta /d},l^{-\eta d},l^{d}\beta^{-1}n^{-1},c_{d}2^{d}n^{-1}\right\}.$$

The solution l=l(n,β) is obtained when $l^{d}{\left(n\right)}^{-\eta /d}=l^{-\eta d}$, or equivalently $l=\lfloor {\left(n\right)}^{\eta /(d^{2}\left(\eta +1\right))}\rfloor$ or when $l^{d}\beta^{-1}n^{-1}=l^{-\eta d}$, which implies $l=\lfloor {\left(\beta \;n\right)}^{1/(d(1+\eta ))}\rfloor$. Substitute this *l* in the bound (A13) to obtain the RHS expression in (13) for d≥2. □

### Appendix A.6.

Our main goal in Section 2.4 was to find proportion α such that the parametric MSE rate depending on the joint density $f_{XY}$ and marginal densities $f_{X},f_{Y}$ is minimized. Recalling the explicit bias bound in (A16), it can be seen that this function will be a complicated function of $f_{XY}$, $f_{X}f_{Y}$ and α. By rearrangement of terms in (A13), we first find an upper bound for Δ in (A16), denoted by $\bar{\Delta }$, as follows:

(A24) $$\begin{array}{c} \bar{\Delta }\left(\alpha ,f_{XY},f_{X}f_{Y}\right)=D\left(n,l_{n},d,\eta \right)+\tilde{D}\left(n,l_{n},d\right)E_{XY}\left[G_{f,n}^{\alpha ,\beta }\left(X,Y\right)\right], \end{array}$$

where $l_{n}:=\left\lfloor n^{\eta /(d^{2}\left(1+\eta \right))}\right\rfloor$. From Appendix A.5 we know that optimal *l* is given by (A23). One can check that for $\alpha \leq 1-n^{(\eta /d)-1}$, the optimal $l=\left\lfloor n^{\eta /(d^{2}\left(1+\eta \right))}\right\rfloor$ provides a tighter bound. In (A24), the constants *D* and $\bar{D}$ are

(A25) $$\begin{array}{c} D\left(n,l_{n},d,\eta \right)=c_{2}l_{n}^{d}n^{-1}+c_{d}2^{d}n^{-1}+c^{\prime }l_{n}^{d}n^{-\eta /d}+cl_{n}^{d}n^{-1/d}+2c_{1}l_{n}^{d-1}n^{1/d-1}+c_{3}l_{n}^{-d\eta }, \end{array}$$

(A26) $$\begin{array}{c} \tilde{D}\left(n,l_{n},d\right)=2+n^{-1}2c^{\prime \prime }\sum_{i=1}^{M}l_{n}\;l_{n}^{d}a_{i}^{-1}+n^{-3/2}2c_{1}^{\prime }\sum_{i=1}^{M}l_{n}\;l_{n}^{d/2}\sqrt{b_{i}}a_{i}^{2}\\ \;+n^{-1}\sum_{i=1}^{M}2n^{-3/2}l_{n}^{-d/2}\frac{\sqrt{b_{i}}}{a_{i}^{2}}{\left(na_{i}l_{n}^{d}+n^{2}a_{i}^{2}\right)}^{1/2}{\left(nb_{i}l_{n}^{d}+n^{2}b_{i}^{2}\right)}^{1/2}. \end{array}$$

And the function $G_{f,n}^{\alpha ,\beta }\left(x,y\right)$ is given as the following:

(A27) $$\begin{array}{c} G_{f,n}^{\alpha ,\beta }\left(x,y\right)=\left(f_{X}\left(x\right)f_{Y}\left(y\right)+\delta_{f}/\left(n\beta \right)\right)(\sqrt{\alpha }f_{XY}\left(x,y\right)\\ +\sqrt{\beta }\left(f_{X}\left(x\right)f_{Y}\left(y\right)+\delta_{f}/\left(\beta n\right)\right)/{\left(\alpha f_{XY}\left(x,y\right)+\beta f_{X}\left(x\right)f_{Y}\left(y\right)\right)}^{2}, \end{array}$$

where $\delta_{f}$ is given in (A15). Next After all still the expression (A27) is complicated to optimize therefore we use the fact that 0≤α,β≤1 to bound the function $G_{f,n}^{\alpha ,\beta }\left(x,y\right)$. Define the set Γ

$$\Gamma :=\left\{\epsilon_{XY}:|\epsilon_{XY}\left(t\right)-\epsilon_{XY}\left(t^{\prime }\right)|\leq \bar{K}{∥t-t^{\prime }∥}_{d}^{\eta }\right\},$$

where

$$\bar{K}=C_{\epsilon }^{U}K\;\left\{C_{XY}^{L}+C_{X}^{L}+C_{Y}^{L}C_{X}^{L}C_{X}^{U}\right\}.$$

Here *K* is the smoothness constant in the Hölder class. Notice that set Γ is a convex set. We bound $\bar{\Delta }$ by

(A28) $$\tilde{\Delta }\left(\alpha ,\epsilon_{XY}\right)=D\left(n,l_{n},d,\eta \right)+\tilde{D}\left(n,l_{n},d\right)\;C_{XY}^{U}\underset{S_{XY}}{\;\int \int \;}{\tilde{G}}_{\epsilon_{XY},n}^{\alpha ,\beta }\left(x,y\right)\;dxdy.$$

Set $C_{n}=C_{XY}^{L}\;n/2$,

(A29) $${\tilde{G}}_{n}^{\alpha ,\beta }\left(\epsilon_{XY}\right)=\frac{\left(\epsilon_{XY}^{-1}\left(x,y\right)+{\left(\beta C_{n}\right)}^{-1})(1+\epsilon_{XY}^{-1}\left(xy\right)+{\left(\beta C_{n}\right)}^{-1}\right)}{{\left(\alpha +\beta \epsilon_{XY}^{-1}\left(x,y\right)\right)}^{2}}.$$

This simplifies to

(A30) $${\tilde{G}}_{n}^{\alpha ,\beta }\left(\epsilon_{XY}\right)=\frac{\left(1+{\left(\beta C_{n}\right)}^{-1}\epsilon_{XY}\right)\left(1+\epsilon_{XY}+{\left(\beta C_{n}\right)}^{-1}\epsilon_{XY}\right)}{{\left(\alpha \epsilon_{XY}+\beta \right)}^{2}}.$$

Under the condition

(A31) $$\frac{2}{C_{n}}\leq \alpha \leq \min\left\{\frac{1}{2}+\frac{1}{2C_{n}},\frac{1}{3}+\frac{2}{3C_{n}}\right\},$$

${\tilde{G}}_{n}^{\alpha ,\beta }\left(\epsilon_{XY}\right)$ is an increasing function in ϵ. Furthermore, for $\alpha \leq \frac{1}{4}$ and

(A32) $$C_{\epsilon }^{L}\leq \epsilon_{XY}\leq \min\left\{C_{\epsilon }^{U},\theta^{U}\left(\alpha \right)\right\},\;\;\;\mathrm{where}\;\;\theta^{U}\left(\alpha \right)=\frac{1-4\alpha +1/C_{n}}{2\alpha },$$

the function ${\tilde{G}}_{n}^{\alpha ,\beta }\left(\epsilon_{XY}\right)$ is strictly concave. Next, to find an optimal α we consider the following optimization problem:

(A33) $$\begin{array}{cc} \min_{\alpha }\;\;\max_{\epsilon_{XY}\in \Gamma } & \tilde{\Delta }\left(\alpha ,\epsilon_{XY}\right)+c_{d}\left(1-\alpha \right)n^{-1}\\ \mathrm{subject}\;\mathrm{to} & C_{\epsilon }^{L}\leq \epsilon_{XY}\leq C_{\epsilon }^{U}, \end{array}$$

here $\epsilon_{XY}=f_{XY}/f_{X}f_{Y}$, $C_{\epsilon }^{U}=C_{XY}^{U}/C_{X}^{L}C_{Y}^{L}$ and $C_{\epsilon }^{L}=C_{XY}^{L}/C_{X}^{U}C_{Y}^{U}$, such that $C_{\epsilon }^{L}\leq 1$. We know that under conditions (A31) and (A32), the function ${\tilde{G}}_{n}^{\alpha ,\beta }$ is strictly concave and increasing in $\epsilon_{XY}$. We first solve the optimization problem:

(A34) $$\begin{array}{cc} \max_{\epsilon_{XY}\in \Gamma } & \underset{S_{XY}}{\;\int \int \;}{\tilde{G}}_{n}^{\alpha ,\beta }\left(\epsilon_{XY}\left(x,y\right)\right)\;dxdy\\ \mathrm{subject}\;\mathrm{to} & \theta_{\epsilon }^{L}\left(\alpha \right)V\left(S_{XY}\right)\leq \underset{S_{XY}}{\;\int \int \;}\epsilon_{XY}\left(x,y\right)dxdy\\ & \;\leq \theta_{\epsilon }^{U}\left(\alpha \right)V\left(S_{XY}\right), \end{array}$$

where

(A35) $$\theta_{\epsilon }^{L}\left(\alpha \right):=C_{\epsilon }^{L},\;\;\;\;\theta_{\epsilon }^{U}\left(\alpha \right):=\min\left\{C_{\epsilon }^{U},\theta^{U}\left(\alpha \right)\right\}.$$

The Lagrangian for this problem is

$$\begin{array}{c} L\left(\epsilon_{XY},\lambda_{1},\lambda_{2}\right)={\;\int \int \;}_{S_{XY}}{\tilde{G}}_{n}^{\alpha ,\beta }\left(\epsilon_{XY}\left(x,y\right)\right)\;dxdy-\lambda_{1}\left({\;\int \int \;}_{S_{XY}}\epsilon_{XY}\left(x,y\right)\;dxdy-\theta_{\epsilon }^{U}\left(\alpha \right)V\left(S_{XY}\right)\right)\\ \;\;\;\;-\lambda_{2}\left(\theta_{\epsilon }^{L}\left(\alpha \right)V\left(S_{XY}\right)-{\;\int \int \;}_{S_{XY}}\epsilon_{XY}\left(x,y\right)\;dxdy\right). \end{array}$$

In this case, the optimum $\epsilon_{XY}^{*}$ is bounded, $\theta_{\epsilon }^{L}\left(\alpha \right)\leq \epsilon_{XY}^{*}\leq \theta_{\epsilon }^{U}\left(\alpha \right),$ and the Lagrangian multiplier $\lambda_{1}^{*},\;\lambda_{2}^{*}\geq 0$ is such that

$$\min_{\lambda_{1},\lambda_{2}\geq 0}\;\;\max_{\epsilon_{XY}\in \Gamma }\;L\left(\epsilon_{XY},\lambda_{1},\lambda_{2}\right)=L\left(\epsilon_{XY}^{*},\lambda_{1}^{*},\lambda_{2}^{*}\right).$$

Set $G_{n}^{\prime }\left(\epsilon_{XY}\right)=\frac{d}{d\epsilon_{XY}}{\tilde{G}}_{n}^{\alpha ,\beta }\left(\epsilon_{XY}\right).$ In view of the concavity of ${\tilde{G}}_{\epsilon_{XY},n}^{\alpha ,\beta }$ and Lemma 1, page 227 in [49], maximizing $L(\epsilon_{XY},\lambda_{1}^{*},\lambda_{2}^{*})$ over $\epsilon_{XY}$ is equivalent to

(A36) $${\;\int \int \;}_{S_{XY}}\left\{G_{n}^{\prime }\left(\epsilon_{XY}^{*}\left(x,y\right)\right)-\left(\lambda_{1}^{*}-\lambda_{2}^{*}\right)\right\}\epsilon_{XY}\left(x,y\right)\;dxdy\leq 0,$$

for all $\theta_{\epsilon }^{L}\left(\alpha \right)\leq \epsilon_{XY}^{*}\leq \theta_{\epsilon }^{U}\left(\alpha \right)$, and

(A37) $${\;\int \int \;}_{S_{XY}}\left\{G_{n}^{\prime }\left(\epsilon_{XY}^{*}\left(x,y\right)\right)-\left(\lambda_{1}^{*}-\lambda_{2}^{*}\right)\right\}\epsilon_{XY}^{*}\left(x,y\right)\;dxdy=0.$$

Denote ${G^{\prime }}_{n}^{-1}$ the inverse function of $G_{n}^{\prime }$. Since $G_{n}^{\prime }$ is strictly decreasing in $\epsilon_{XY}^{*}$ (this is because ${\tilde{G}}_{n}^{\alpha ,\beta }\left(\epsilon_{XY}\right)$ is strictly concave, so that ${G^{\prime }}_{n}^{-1}$ is continuous and strictly decreasing in $\epsilon_{XY}^{*}$). From (A36) and (A37), we see immediately that on any interval $\theta_{\epsilon }^{L}\left(\alpha \right)\leq \epsilon_{XY}^{*}\leq \theta_{\epsilon }^{U}\left(\alpha \right)$, we have $\epsilon_{XY}^{*}={G^{\prime }}_{n}^{-1}\left(\lambda_{1}^{*}-\lambda_{2}^{*}\right)$. We can write then

$$G_{n}^{\prime }\left(\theta_{\epsilon }^{U}\left(\alpha \right)\right)\leq \lambda_{1}^{*}-\lambda_{2}^{*}\leq G_{n}^{\prime }\left(\theta_{\epsilon }^{L}\left(\alpha \right)\right),$$

and $\lambda_{1}^{*},\lambda_{2}^{*}\geq 0$. Next, we find the solution of

$$\min_{\lambda_{1},\lambda_{2}\geq 0}\;\;{\bar{G}}_{n}^{\alpha ,\beta }\left(\lambda_{1},\lambda_{2}\right),\;\;\;\mathrm{where}$$

$$\begin{array}{c} {\bar{G}}_{n}^{\alpha ,\beta }\left(\lambda_{1},\lambda_{2}\right)=V\left(S_{XY}\right)\left\{{\tilde{G}}_{n}^{\alpha ,\beta }\left({G^{\prime }}_{n}^{-1}\left(\lambda_{1}-\lambda_{2}\right)\right)-\left(\lambda_{1}-\lambda_{2}\right){G^{\prime }}_{n}^{-1}\left(\lambda_{1}-\lambda_{2}\right)+\lambda_{1}\theta_{\epsilon }^{U}\left(\alpha \right)-\lambda_{2}\theta_{\epsilon }^{L}\left(\alpha \right)\right\}. \end{array}$$

The function ${\bar{G}}_{n}^{\alpha ,\beta }\left(\lambda_{1},\lambda_{2}\right)$ is increasing in $\lambda_{1}$ and $\lambda_{2}$, and therefore it takes its minimum at $\left(\lambda_{1}^{*},\lambda_{2}^{*}\right)=\left(G_{n}^{\prime }\left(\theta_{\epsilon }^{U}\left(\alpha \right)\right),0\right)$. This implies that $\epsilon_{XY}^{*}=\theta_{\epsilon }^{U}\left(\alpha \right)$. Returning to our primary minimization over α:

(A38) $$\begin{array}{cc} \underset{\alpha }{\min\;\;} & \tilde{\Delta }\left(\alpha ,\epsilon_{XY}^{*}\right)+c_{d}\left(1-\alpha \right)n^{-1}\\ \mathrm{subject}\;\mathrm{to} & \alpha_{0}^{L}\leq \alpha \leq \alpha_{0}^{U}, \end{array}$$

where $\alpha_{0}^{L}=\frac{2}{C_{n}}$ and $\alpha_{0}^{U}=\min\left\{\frac{1}{4},1-n^{\eta /d-1}\right\}$. We know that $\frac{1}{4}\leq \frac{1}{3}+\frac{2}{3C_{n}}$ and $\frac{1}{4}\leq \frac{1}{2}+\frac{1}{2C_{n}}$, therefore the condition below

$$\frac{2}{C_{n}}\leq \alpha \leq \min\left\{\frac{1}{4},1-n^{\eta /d-1}\right\},$$

implies the constraint $\alpha_{0}^{L}\leq \alpha \leq \alpha_{0}^{U}$. Since the objective function (A38) is a complicated function in α, it is not feasible to determine whether it is a convex function in α. For this reason, let us solve the optimization problem in (A38) in a special case when $C_{\epsilon }^{U}\leq \theta^{U}\left(\alpha \right)$. This implies $\epsilon_{XY}^{*}=C_{\epsilon }^{U}$. Under assumption $C_{\epsilon }^{U}$ the objective function in (A38) is convex in α. Also, the case $C_{\epsilon }^{U}\leq \theta^{U}\left(\alpha \right)$ is equivalent to $\alpha \leq \frac{1+1/C_{n}}{4+2C_{\epsilon }^{U}}$. Therefore, in the optimization problem we have constraint

$$\frac{2}{C_{n}}\leq \alpha \leq \min\left\{\frac{1}{4},\frac{1+1/C_{n}}{4+2C_{\epsilon }^{U}},1-n^{\eta /d-1}\right\}.$$

We know that $\tilde{\Delta }\left(\alpha ,\epsilon_{XY}^{*}\right)+c_{d}\left(1-\alpha \right)n^{-1}$ is convex over $\alpha \in [\alpha_{0}^{L},\alpha_{0}^{U}]$. Therefore, the problem becomes ordinary convex optimization problem. Let $\tilde{\alpha }$, ${\tilde{\lambda }}_{1}$ and ${\tilde{\lambda }}_{2}$ be any points that satisfy the KKT conditions for this problem:

(A39) $$\begin{array}{c} \alpha_{0}^{L}-\tilde{\alpha }\leq 0,\;\;\;\tilde{\alpha }-\alpha_{0}^{U}\leq 0,\;\;\;{\tilde{\lambda }}_{1},{\tilde{\lambda }}_{2}\geq 0,\\ {\tilde{\lambda }}_{1}\left(\alpha_{0}^{L}-\tilde{\alpha }\right)=0,\;\;\;{\tilde{\lambda }}_{2}\left(\tilde{\alpha }-\alpha_{0}^{U}\right)=0,\\ \frac{d}{d\alpha }\left(\tilde{\Delta }\left(\tilde{\alpha },\epsilon_{XY}^{*}\right)+c_{d}\left(1-\tilde{\alpha }\right)\;n^{-1}\right)-{\tilde{\lambda }}_{1}+{\tilde{\lambda }}_{2}=0. \end{array}$$

Recall Ξ(α) from (20):

$$\begin{array}{c} \Xi \left(\alpha \right)=\frac{d}{d\alpha }\left(\tilde{\Delta }\left(\alpha ,\epsilon_{XY}^{*}\right)+c_{d}\left(1-\alpha \right)n^{-1}\right), \end{array}$$

where $\tilde{\Delta }$ is given in (A28). So, the last condition in (A39) becomes $\Xi \left(\tilde{\alpha }\right)={\tilde{\lambda }}_{1}-{\tilde{\lambda }}_{2}$. We then have

$$\alpha_{0}^{L}\leq \Xi^{-1}\left({\tilde{\lambda }}_{1}-{\tilde{\lambda }}_{2}\right)\leq \alpha_{0}^{U},$$

where $\Xi^{-1}$ is inverse function of Ξ. Since $\alpha_{0}^{L}\neq \alpha_{0}^{U}$, at least one of ${\tilde{\lambda }}_{1}$ or ${\tilde{\lambda }}_{2}$ should be zero:

- ${\tilde{\lambda }}_{1}=0$, ${\tilde{\lambda }}_{2}\neq 0$. Then $\tilde{\alpha }=\alpha_{0}^{U}$ and implies ${\tilde{\lambda }}_{2}=-\Xi \left(\alpha_{0}^{U}\right)$. Since ${\tilde{\lambda }}_{2}>0$, so this leads to $\Xi (\alpha_{0}^{U})<0$.
- ${\tilde{\lambda }}_{2}=0$, ${\tilde{\lambda }}_{1}\neq 0$. Then $\tilde{\alpha }=\alpha_{0}^{L}$ and implies ${\tilde{\lambda }}_{1}=\Xi \left(\alpha_{0}^{L}\right)$. We know that ${\tilde{\lambda }}_{1}>0$, hence $\Xi (\alpha_{0}^{L})>0$.
- ${\tilde{\lambda }}_{1}=0$, ${\tilde{\lambda }}_{2}=0$. Then $\tilde{\alpha }=\Xi^{-1}\left(0\right)$ and so $\alpha_{0}^{L}\leq \Xi^{-1}\left(0\right)\leq \alpha_{0}^{U}$.

Consequently, by following the behavior of Ξ(α) with respect to $\alpha_{0}^{L}$ and $\alpha_{0}^{U}$, we can often find the optimal $\tilde{\alpha }$, ${\tilde{\lambda }}_{1}$ and ${\tilde{\lambda }}_{2}$. For instance, if Ξ(α) is positive for all $\alpha \in [\alpha_{0}^{L},\alpha_{0}^{U}]$ then we conclude that $\tilde{\alpha }=\alpha_{0}^{L}$.
